# Scalable and Multifunctional PAN‐MXene Composite Fibers for Thermal Management, Photothermal Conversion, Energy Harvesting, and Sensing for Wearable Applications

**DOI:** 10.1002/adma.202522098

**Published:** 2025-12-30

**Authors:** Ahmadreza Moradi, Piotr K. Szewczyk, Urszula Stachewicz

**Affiliations:** ^1^ Faculty of Metals Engineering and Industrial Computer Science AGH University of Krakow Krakow Poland

**Keywords:** electrospinning, multifunctional yarns, MXene, polyacrylonitrile (PAN), smart textiles, thermally conductive fibers, triboelectric energy harvesting

## Abstract

Developing multifunctional materials that combine efficient heat conduction, energy harvesting, sensing capability, and flexibility is crucial for next‐generation portable and wearable electronics. Here, exploiting the remarkable properties of Ti_3_C_2_T_x_ MXene nanosheets, multifunctional polyacrylonitrile (PAN)‐MXene nanofibers and yarns are fabricated via a straightforward and scalable electrospinning process. Incorporation of MXenes enhances the thermal conductivity of individual PAN nanofibers, as measured by scanning thermal microscopy, and greatly increases the heat conduction capacity of composite yarns, showing a ∼22°C higher surface temperature recorded by infrared thermography. The composite nanofibers also exhibit strong passive heating capability, rapidly reaching up to 60°C under infrared irradiation. Furthermore, MXenes elevate the tribo‐negative character of PAN nanofibers, decreasing their surface potential to −360 mV and yielding a high triboelectric power density of 432.7 mW m^−2^, approximately 25% higher than pristine PAN. Moreover, the produced composite yarns demonstrate reliable tactile‐sensing performance, detecting forces as low as 0.1 N. Altogether, these flexible and durable PAN‐MXene structures provide a promising route toward sustainable and energy‐autonomous electronic textiles, offering new opportunities in wearable electronics, soft robotics, and smart sensing systems.

## Graphical abstract

Electrospun PAN‐MXene nanofibers and yarns integrate enhanced thermal conductivity, photothermal conversion, and triboelectric energy harvesting within a flexible architecture. Interconnected MXene networks promote efficient phonon transport, while their surface chemistry strengthens tribo‐negative behavior, enabling a high power density of 432.7 mW m^−2^ and a sensitive tactile response for multifunctional smart textile applications.

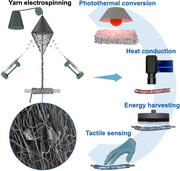

## 1 Introduction

The rapid miniaturization of electronic components and the expansion of flexible and wearable devices have created an urgent demand for materials capable of efficient thermal management. This includes both localized heat dissipation in compact circuits and adaptive thermal comfort in wearable systems [1, 2]. Conventional cooling methods, such as metallic heat sinks or active cooling systems, are impractical for small, lightweight, and flexible devices. This makes thermally conductive polymer materials a compelling solution to combine flexibility with effective heat removal in the next generation of wearable and miniaturized electronics [3, 4, 5].

To overcome the intrinsically low thermal conductivity of polymers (< 0.5 W m^−1^ K^−1^), incorporating thermally conductive fillers has become the most practical and scalable approach [6, 7]. Carbon‐based, ceramic, and metallic fillers have been widely employed to form continuous phonon‐transport networks within polymer matrices, markedly improving their heat conduction capability [8, 9, 10]. Despite these advances, the progress of polymer composite development still lags behind the pace of emerging electronic technologies. As devices become smaller, lighter, and more multifunctional, materials optimized only for heat conduction must often be integrated with additional components for sensing, heating, or energy harvesting [11, 12]. Such multi‐component assemblies increase fabrication complexity and cost, hinder further miniaturization, and can compromise flexibility, which are key requirements for wearable and textile‐integrated technologies.

A promising approach to address these challenges is to design multifunctional composites, where a single material simultaneously provides multiple properties such as high thermal conductivity, electrical response, and energy storage capability. This can be achieved by incorporating multiple fillers, each contributing distinct functionalities [13, 14, 15]. However, the combination of dissimilar fillers often introduces additional challenges. High thermal conductivity generally requires large filler contents, which already challenge uniform dispersion [16, 17]. Adding additional fillers can intensify this problem, not only by increasing the overall filler concentration but also by introducing disparities in particle size, density, or surface energy that promote phase segregation [18, 19]. In many cases, the formation of competing or disconnected filler networks can limit the expected synergistic enhancement and lead to anisotropic behavior [20, 21]. Moreover, processing difficulties, such as limited solvent compatibility, increased viscosity, and reduced scalability, further constrain fabrication. Thus, achieving true multifunctionality through simple additive stacking demands precise interface engineering, controlled filler dispersion, and deliberate network design to establish cooperative interactions among multiple filler types.

A more practical route to achieve multifunctionality is to employ fillers that inherently combine several properties within one material. This strategy minimizes interfacial complexity, simplifies processing, and enables balanced optimization of performance and cost. Among such materials, MXenes, a family of 2D transition‐metal carbides, nitrides, and carbonitrides, stand out as exceptional candidates. The archetypal MXene, Ti_3_C_2_T_x_ (where T_x_ denotes surface terminations such as ‐O, ‐OH, and ‐F), exhibits metallic‐level electrical conductivity, high in‐plane thermal conductivity, excellent volumetric capacitance, and tunable surface chemistry that enables strong bonding with polar polymers [22, 23, 24, 25]. Its hydrophilic nature facilitates dispersion in aqueous and polar media, allowing the preparation of stable polymer‐MXene formulations. Previous studies have shown that MXene‐based composites can concurrently enhance thermal and electrical conductivity while providing efficient electromagnetic interference (EMI) shielding and multifunctional energy‐conversion properties, including Joule heating, photothermal, and triboelectric responses [26, 27, 28, 29]. This makes them highly promising for wearable and flexible electronics. However, many reported systems rely on multistep or template‐assisted fabrication methods, such as layer‐by‐layer assembly or vacuum filtration, which hinder large‐scale production.

Electrospinning offers a versatile and scalable method to produce composite fibrous structures in a single manufacturing step [30, 31, 32, 33]. It enables the incorporation of high filler concentrations with uniform distribution, critical for thermally conductive composites [34, 35]. The strong electric field and elongational forces during spinning promote polymer chain alignment and orient fillers along the fiber axis, fostering continuous and interconnected thermal pathways with reduced interfacial thermal resistance [36, 37, 38, 39, 40]. These features significantly enhance phonon transport across the composite. Owing to its adaptability in producing fibers, mats, and yarns, electrospinning is particularly appealing for wearable applications, where breathability, flexibility, low thickness, and seamless textile integration are critical design requirements [41, 42, 43, 44].

**FIGURE 1:**
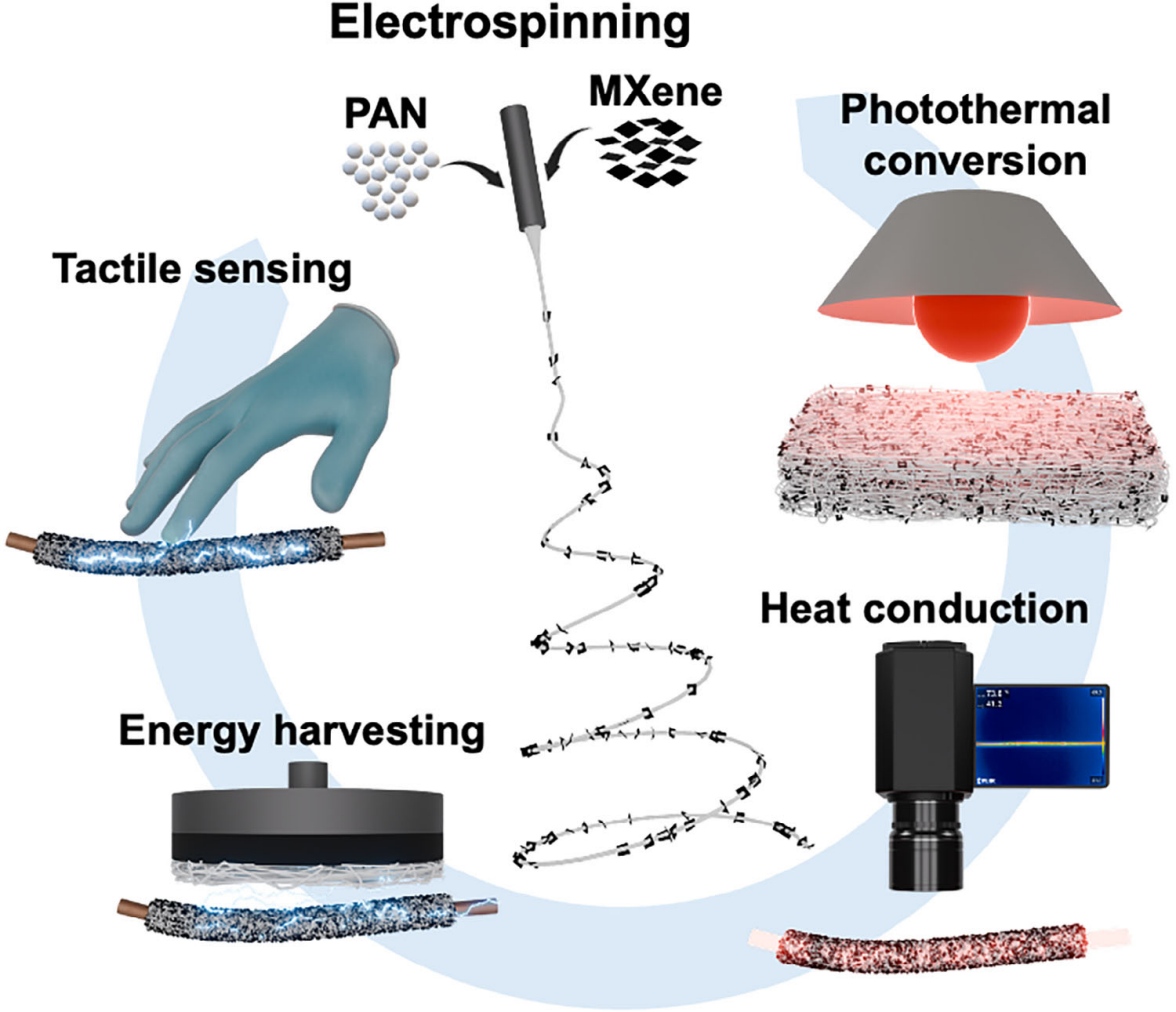
Conceptual schematic illustrating the multifunctional design strategy of electrospun PAN‐MXene composites, integrating thermal, photothermal, and triboelectric functionalities that enable efficient heat dissipation, energy harvesting, and tactile sensing in smart textile applications.

In this context, polyacrylonitrile (PAN) serves as a highly suitable polymer matrix for electrospun composites. PAN exhibits excellent thermal stability, mechanical robustness, and good chemical resistance under conditions relevant to wearable textiles and thermal‐management applications, and is widely used as a precursor for carbon fibers [45, 46, 47]. Its nitrile groups form strong interactions with the surface terminations of MXene, improving interfacial adhesion and charge transfer within the composite [48, 49, 50]. Despite extensive research on electrospun PAN‐based systems in which MXene is used as an active component within stabilized or carbonized architectures [51, 52], the impact of MXene incorporation on the thermal conductivity and multifunctional performance of electrospun PAN structures remains largely unexplored. More importantly, existing studies typically address individual functions or rely on complex architectures, whereas an integrable and scalable electrospun yarn platform that combines multiple functionalities has not yet been demonstrated. Therefore, in this work, we demonstrate the fabrication of electrospun PAN‐MXene nanofibers that integrate enhanced thermal conductivity, photothermal conversion, and triboelectric energy‐harvesting functionality. The incorporation of multilayer Ti_3_C_2_T_x_ MXene nanoflakes into the PAN matrix yields uniform, interconnected nanofibrous networks that preserve structural integrity and maintain excellent mechanical flexibility. Differential scanning calorimetry (DSC) confirmed improved thermal stability in the composite nanofibers, while strong photothermal conversion was observed under infrared (IR) illumination. Scanning thermal microscopy (SThM) and IR thermography further verified a significant enhancement in heat transport across multiple length scales, from the nanoscale to the macroscopic level. In addition, Kelvin probe force microscopy (KPFM) revealed that MXene incorporation lowers the surface potential of PAN, rendering the fibers more tribo‐negative. Electrospun coating yarns composed of these nanofibers exhibited elevated triboelectric power generation and force sensitivity when integrated into tactile‐sensing arrays. Altogether, these results position electrospun PAN‐MXene composites as a scalable, multifunctional platform for lightweight, flexible, and thermally efficient materials in next‐generation electronic and wearable technologies. Figure 1 represents the core concept of this research.

## 2 Results and Discussion

### 2.1 Morphology and Chemical Composition of Electrospun Fibers

**FIGURE 2:**
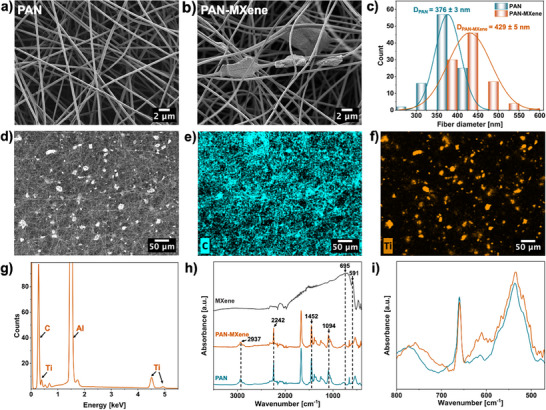
SEM micrographs illustrating the morphology of electrospun nanofibers: (a) PAN and (b) PAN‐MXene; (c) fiber diameter distribution curves with the average fiber diameter for PAN and PAN‐MXene nanofibers. Data are presented as mean ± SE (*n* = 100 fibers per sample). (d) SEM micrographs of PAN‐MXene nanofibers in lower magnification, and (e–g) corresponding EDS mapping images of C and Ti elements along with the EDS spectrum. (h) ATR‐FTIR spectra of electrospun PAN nanofibers, PAN‐MXene nanofibers, and MXene powder; (i) magnified view of the fibers’ spectra in the 500–800 cm^−1^ region without the vertical offset.

PAN nanofibers and PAN nanofibers containing 50 wt.% multilayer MXene nanoflakes (with respect to polymer content) were produced by electrospinning under identical processing conditions. The MXene concentration was optimized to achieve the highest filler loading while still maintaining a stable and continuous fiber production process. This composition was selected with the aim of achieving sufficient filler network density and approaching the percolation threshold, which is considered critical for enhancing the thermal and electrical functionalities of composite fibers, as one of the primary objectives of this work. Figure 2a,b represents the morphology of the resulting randomly oriented nanofibers. In the composite nanofibers, the presence of multilayer MXene nanoflakes is clearly visible among the nanofibers, in contrast to the smooth morphology of pure PAN nanofibers. The incorporation of MXene slightly increased the average fiber diameter from 376 ± 3 nm for PAN to 429 ± 5 nm for PAN‐MXene, see Figure 2c. This change is attributed to the higher viscosity of the PAN‐MXene solution, which reduces jet elongation during electrospinning and results in thicker fibers [53, 54]. Elemental mapping of Ti (Figure 2d–g) confirms the widespread distribution of MXene sheets throughout the fiber network, demonstrating the effectiveness of electrospinning for producing composites with high filler contents. However, unlike nanofillers that are smaller than the fiber diameter and can be readily embedded within the individual fibers, the large size of multilayer MXene nanosheets, characterized by an average equivalent circle diameter (ECD) of 7 ± 0.2 µm, combined with the difficulty in delaminating the stacked layers (Figure S1), hindered their uniform incorporation within individual fibers. Consequently, the MXene nanosheets predominantly appear as individually dispersed multilayered flakes with occasional localized agglomerates, located mainly on the fiber surfaces, protruding from them, or bridging between adjacent fibers. As a result, percolation pathways are more likely to form at MXene‐rich fiber junctions, where structural connections can form, rather than within individual fibers.

To investigate the effect of MXene incorporation on the chemical structure of PAN nanofibers, ATR‐FTIR analysis was performed on the nanofibers and MXene nanoflakes (Figure 2h). In the spectrum of pure PAN nanofibers, the absorption band at 2937 cm^−1^ is assigned to –CH_2_– stretching vibrations, while the sharp peak at 2242 cm^−1^ corresponds to the –C≡N stretching of acrylonitrile units in the polymer backbone. Additional absorptions at 1452 and 1094 cm^−1^ arise from –CH_2_– bending modes [55, 56, 57].  The MXene nanoflakes exhibit broad absorptions at 695 and 591 cm^−1^, attributed to Ti–O stretching vibrations [58, 59, 60]. However, in the spectrum of PAN‐MXene nanofibers, these distinct MXene peaks are not apparent, which has also been reported in some previous studies [57, 61, 62]. Nevertheless, as shown in the magnified spectra in Figure 2i, the incorporation of MXene led to an increase in absorption intensity of composite fibers within the 500–800 cm^−1^ region, where the characteristic MXene absorptions are observed.

### 2.2 Mechanical Properties and Thermal Analysis

**FIGURE 3:**
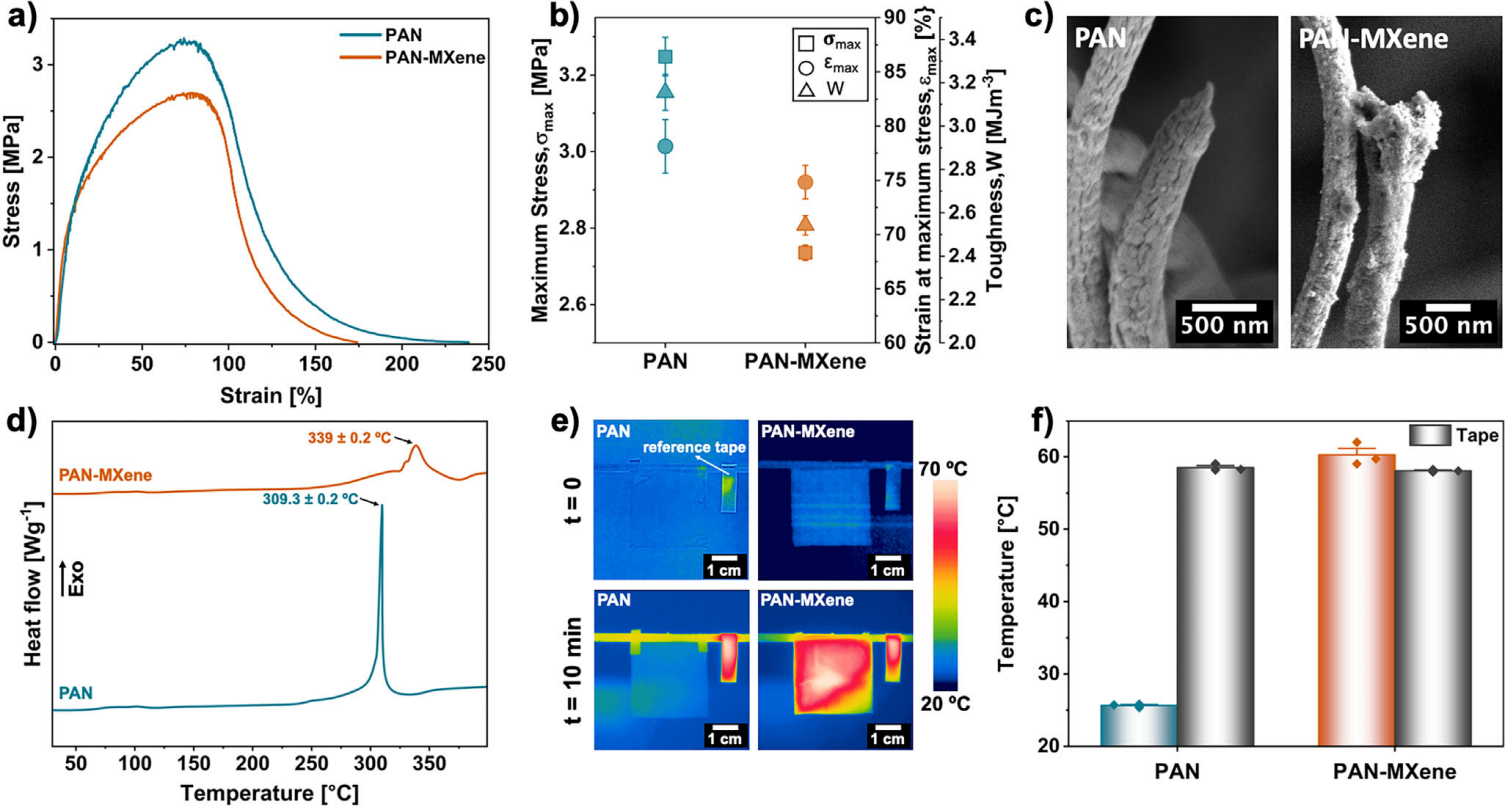
(a) Stress–strain curves of the electrospun PAN and PAN‐MXene nanofiber mats, and (b) summary of their mechanical properties. Error bars represent SE (*n* = 3). (c) SEM micrographs of fractured PAN and PAN‐MXene nanofibers after mechanical test. (d) DSC heating thermograms of PAN and PAN‐MXene nanofibers. Data are presented as mean ± SE (*n* = 3). (e) Thermal images of PAN and PAN‐MXene nanofiber mats irradiated by an IR lamp, and (f) column chart presenting the average surface temperatures of the samples and their corresponding reference tape. Error bars represent SE (*n* = 3), and individual data points indicate independent measurements.

To assess suitability for flexible and wearable applications, the mechanical properties of the electrospun nanofibers were evaluated using the tensile test. Figure 3a presents representative stress–strain curves for randomly oriented nanofibers. The PAN nanofibers showed good mechanical performance during the measurements, reaching a maximum stress (σ_max_) of ∼ 3.2 MPa and a strain at failure (ε_f_) above 200%. Upon incorporation of a high concentration of MXene nanoflakes, the mechanical performance of the nanofibers decreased. This degradation is attributed primarily to the presence of rigid multilayer MXene flakes and their tendency to form agglomerates at high filler loading, which act as stress concentrators and promote premature damage under tensile loading [63,64]. As a result, the σ_max_ decreased by ∼ 17% to 2.74 MPa for the PAN‐MXene nanofibers, see Figure 3b and Table S1. Consistent with this interpretation, SEM micrographs acquired after tensile testing (Figure 3c) reveal that fracture in the composite nanofibers initiates preferentially near MXene‐rich regions.  Similar agglomeration‐induced degradation at high MXene content has been widely reported in polymer/MXene composite fibers, where weak cohesion within agglomerates limits effective stress transfer and accelerates failure [48,61]. The presence of MXene had little effect on the strain at maximum stress (ε_max_), indicating that up to this deformation level, fiber reorientation and sliding within the randomly oriented network were not significantly hindered. In contrast, ε_f_ decreased by ∼ 26% (Figure 3b; Table S1), indicating reduced ductility at higher strains. This behavior can be attributed to the limited plastic deformation of the ceramic MXene flakes combined with agglomeration, which restricts further fiber mobility and promotes localized fracture. Comparable trends, where initial reinforcement is followed by mechanical degradation at higher MXene loadings, have been reported in other electrospun polymer/MXene systems [65,66]. As a result of the combined reductions in tensile strength and ductility, the toughness decreased from 3.16 ± 0.08 to 2.54 ± 0.04 MJ m^−3^ for the PAN‐MXene samples. While these reductions reflect the expected trade‐off associated with incorporating a high content of functional filler, the composite nanofibers still retain high stretchability and practical strength levels, falling within the range reported for electrospun networks used in applications such as smart textiles, thermal management, and wearable electronics [67, 68, 69, 70]. To alleviate this trade‐off, enhancing the interfacial adhesion between the MXene nanosheets and the PAN matrix, through strategies such as MXene surface modification, may offer an effective approach. Limited matrix‐filler interaction is likely a contributing factor to the reduced mechanical integrity observed at such high filler content. Although no direct studies have examined this effect in PAN‐MXene systems, several reports have demonstrated that MXene surface functionalization via silanization, organic–inorganic grafting, or MOF coating can improve mechanical performance [71, 72, 73, 74]. Such modifications can strengthen interfacial bonding and reduce aggregation, making them a promising strategy for improving the mechanical performance of PAN‐MXene composites in future developments.

DSC measurements were performed to assess the impact of MXene on the thermal behavior of the nanofibers. As shown in Figure 3d, no melting peaks were detected for the samples. The DSC curve of PAN displays a sharp exothermic peak centered at 309.3°C ± 0.2°C, attributed to the cyclization of nitrile groups on PAN molecular chains under an oxygen‐free atmosphere. This reaction begins at 303.4°C ± 0.1°C and ends at 310.3°C ± 0.3°C. During cyclization, adjacent nitrile groups on the PAN backbone react with each other, converting the linear structure of PAN into a ladder‐like structure with conjugated double bonds and aromatic‐like rings [75,76]. Notably, the addition of MXene had a pronounced effect on the cyclization of the PAN nanofibers. In the representative DSC curve for PAN‐MXene, the exothermic peak shifts by ∼ 30°C to a higher temperature (339°C ± 0.2°C). The peak also becomes broader, with the reaction starting at 326.9°C ± 0.2°C and finishing at 353.4°C ± 0.3°C. In addition, the cyclization enthalpy (ΔH) decreases by ∼ 18%, from 452.3 ± 3.7 to 374.36 ± 6.3 Jg^−1^. Together with the broader temperature window, this indicates a more distributed heat release (lower average exothermic rate), which mitigates rapid self‐heating during the reaction. This lowers the likelihood of PAN chain scission and supports safer, more controllable stabilization for carbon‐fiber production, where scale‐dependent runaway exotherms are a known risk [77]. One plausible reason behind this behavior is that the near‐percolation, highly thermally conductive MXene network acts as a heat sink during the measurement, conducting the exotherm's heat away [78]. Consequently, with reduced self‐heating, a higher furnace temperature is needed to achieve the same reaction rate, yielding a broader peak at a higher temperature. In addition, the interfacial restriction due to adsorption and confinement of PAN chains at MXene surfaces, reduces chain mobility and effective nitrile proximity, which slows the cyclization process and further shifts and widens the peak [79]. Furthermore, FTIR analysis after the DSC measurements (Figure S2) shows no evidence of new PAN‐MXene chemical bonding, indicating that detectable chemical interactions between PAN's nitrile groups and MXene surface terminations do not contribute to the observed cyclization shift. Consistent with these findings, Mokhtari et al. observed that incorporating MXene into PAN nanofibers reduced the exothermic enthalpy, with larger reductions at higher MXene contents, while the peak temperature remained approximately constant across samples [80]. It should be noted that their electrospun structures contained much less MXene (∼ 8 wt.%), and the MXene loading for the dip‐coated samples was not reported. As indicated by the DSC results for PAN and PAN‐MXene, no melting peak was detected, reflecting the high thermal stability of the nanofibers across the examined range. In particular, the composite nanofibers tolerated heating up to 327°C without an observable structural change, which is well above the typical operating temperatures used in many heat‐transfer applications. Furthermore, the glass transition (T_g_, expected near ∼ 100°C) was not detected, likely due to the 10°C min^−1^ heating rate employed, consistent with several prior reports [81,82].

MXenes are distinguished by their excellent photothermal conversion capability. Their metallic to semi‐metallic conductivity and layered structure enable strong broadband absorption of incident photons, with the absorbed energy efficiently dissipated through non‐radiative electron‐phonon interactions that generate heat. In addition, their high density of free charge carriers gives rise to localized surface plasmon resonnances (LSPRs) in the visible‐NIR region, further enhancing photon absorption. Strong UV absorption can also result from interband transitions. Collectively, these features ensure broad‐spectrum light harvesting [83, 84, 85]. Shi et al. demonstrated this effect by depositing MXene on patterned TPU electrospun membranes, where the surface temperature increased from 20°C to 32.4°C after 100 s of light irradiation, whereas pristine TPU showed no meaningful photothermal response and remained at ∼ 22°C [86]. Chang et al. further showed that cellulose/MXene composite fibers containing 8 wt.% MXene reached 62.1°C within 2 min under simulated solar irradiation (1000 Wm^−2^), compared to only 34.9°C for pure cellulose fibers [87]. In the present work, we evaluated the photothermal performance of the electrospun PAN‐MXene nanofibers. Thermal images of nanofiber mats before and after 10 min of IR irradiation (Figure 3e) reveal a clear temperature contrast between pristine and composite samples. To minimize heat dissipation through conduction and ensure a proper exposure to IR radiation, the mats were suspended vertically on a low‐absorbing plastic stand using small tape strips, with a reference tape placed adjacent to the samples under identical conditions. As shown in Figure 3f, the composite nanofibers reached an equilibrium surface temperature of 60.3°C ± 0.9°C, nearly 35°C higher than pristine PAN nanofibers (25.6°C ± 0.1°C), which remained close to room temperature (22°C–24°C). These results demonstrate the excellent photothermal conversion efficiency of PAN‐MXene nanofibers and underline their potential in diverse applications, including thermal management, energy harvesting, and sensing technologies.

### 2.3 Heat Conduction Performance of the Nanofibers

**FIGURE 4:**
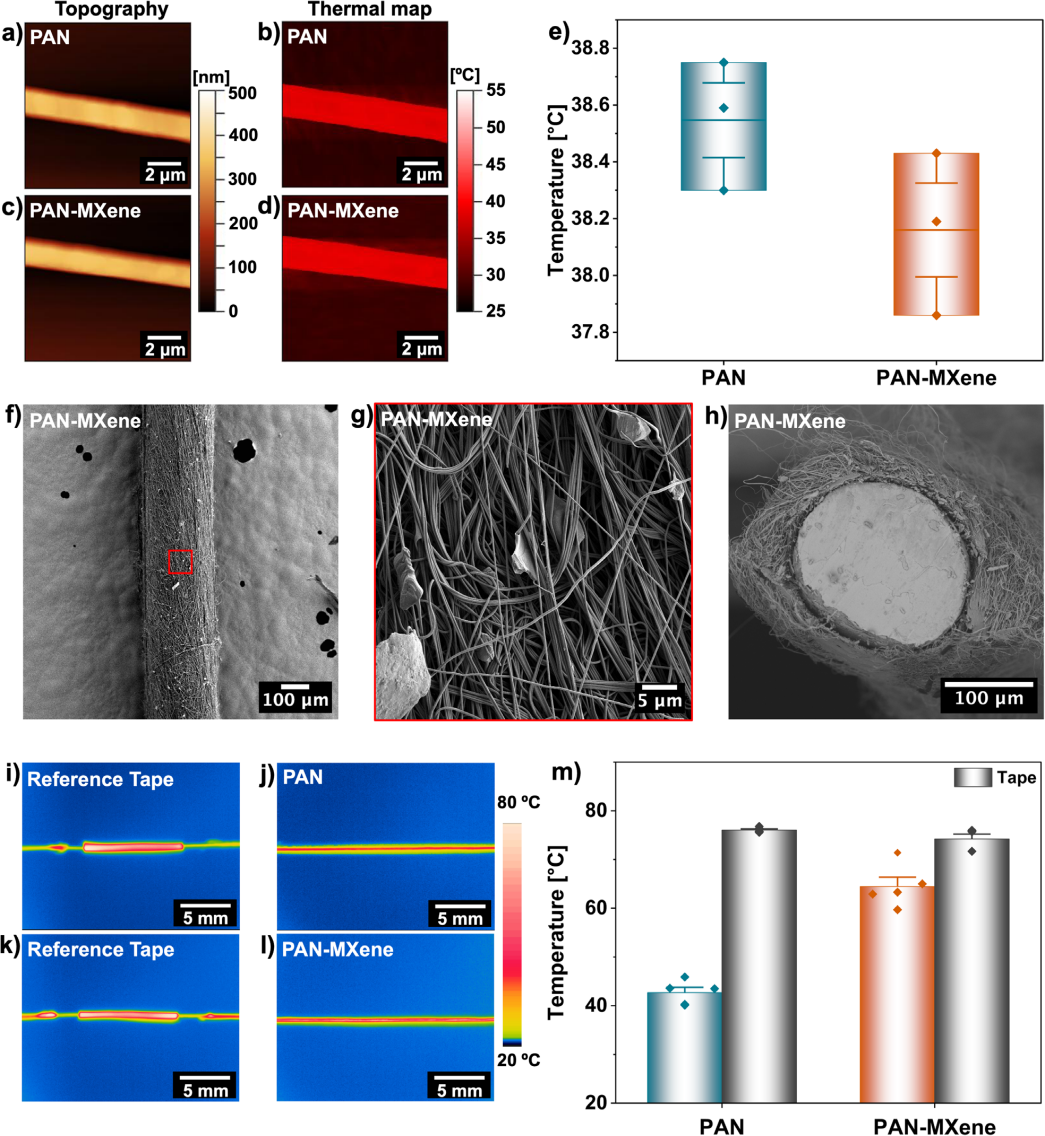
The topography and thermal conductivity maps of (a,b) PAN and (c,d) PAN‐MXene nanofibers, measured by SThM. (e) Box charts presenting the average temperature of the tip on the individual nanofibers. Boxes represent the 25%–75% range, the central line indicates the mean value, whiskers represent SE, and individual points correspond to independent measurements (*n* = 3). (f–h) SEM micrographs of the surface morphology and cross‐section of PAN‐MXene yarn coated on a resistive wire. Thermal images of (i,j) the reference tape and PAN yarn, and (k,l) the reference tape and PAN‐MXene yarn on a heated resistive wire. (m) Column chart showing the average surface temperature of the yarns and their corresponding reference tapes. Error bars represent SE (*n* = 5), and individual data points denote independent measurements.

In this study, the thermal conductivity of individual nanofibers was evaluated using SThM. Measurements were carried out in the thermal conductivity contrast mode, where the temperature of the heated tip is inversely correlated with the thermal conductivity of the underlying material. Materials with higher thermal conductivity dissipate heat more effectively from the tip, leading to a lower recorded probe temperature [88, 89, 90]. Figure 4a–d presents representative topography images alongside the corresponding thermal maps for individual PAN and PAN‐MXene nanofibers deposited on ITO glass substrates, recorded with a probe heated to 50°C above the ambient temperature. Due to measurement challenges at PAN/MXene junctions, SThM measurements were performed only on fiber segments. As depicted in Figure 4e, the average temperature of the tip was ∼ 0.4°C lower when in contact with the composite nanofibers compared to pristine PAN, indicating the enhanced thermal conductivity imparted by the incorporation of MXene nanosheets. It should be mentioned that the ITO background underlying PAN nanofibers exhibited higher thermal conductivity than the substrate beneath the PAN‐MXene nanofibers, as indicated by its lower probe temperature, see Figure S3a. This factor could bias the measurements in favor of pristine PAN; nevertheless, the composite nanofibers consistently demonstrated superior conductivity relative to their counterparts. These findings were further supported by SThM measurements in spectroscopy mode, performed over randomly selected line scans across multiple nanofibers, which reproduced the same trend of higher thermal conductivity in the composites compared to PAN nanofibers (Figure S3b,c).

To investigate the macroscopic thermal conduction performance of the nanofibers and assess their potential in thermal management applications, electrospun coating yarns were fabricated using equipment with a dedicated yarn electrospinning module. In this setup, precursor solutions were electrospun simultaneously from positively and negatively charged nozzles toward a rotating vortex collector. A resistive wire was continuously passed through the center of the collector, enabling the nanofibers to deposit and wrap uniformly around its surface. Afterward, the coated yarns were collected on a mandrel; see Figure S4a and Video S1. SEM micrographs of the resulting yarns (Figure 4f–h; Figure S2b–d) reveal that the nanofibers were partially aligned along the resistive wire and provided a consistent coating over its surface. The yarns exhibited an overall diameter, including the core wire, of 241 ± 4 µm for PAN and 246 ± 2 µm for PAN‐MXene (Figure S4e). Moreover, the morphology of nanofibers within the yarns was comparable to those produced by single nozzle electrospinning, with slightly lower average fiber diameters of 303 ± 4 nm for PAN and 340 ± 5 nm for PAN‐MXene, as illustrated in Figure S4f. The embedded resistive wire was then employed as a Joule‐heating source to assess the thermal conductivity of the yarns. Voltage was applied via an adjustable power supply to increase the wire temperature, and the thermal response of the coated yarns was recorded in real time using infrared thermography. A standard reference tape was placed alongside the samples to calibrate emissivity and ensure consistency across measurements. For all tests, the applied voltage was adjusted such that the maximum surface temperature of the reference tape reached 80°C. Representative thermal images of the PAN and PAN‐MXene yarns with their corresponding reference tapes are shown in Figure 4i–l. Under identical conditions, the composite yarns reached an average surface temperature of 64.5°C ± 1.9°C, approximately 22°C higher than that of the PAN yarns (42.7°C ± 1.1°C), see Figure 4m. These results clearly demonstrate the superior heat conduction capability of the PAN‐MXene yarns, enabled by the formation of efficient thermal pathways through the MXene nanosheets. Acting as conductive bridges between adjacent nanofibers, the nanosheets facilitated phonon transport across the porous architecture. The observed enhancement is consistent with prior reports on MXene‐based polymer composites. For example, Kang et al. demonstrated that incorporating only 1 wt.% Ti_3_C_2_ MXene into epoxy increased the thermal conductivity by ∼ 141% (from 0.243 to 0.578 W m^−1^ K^−1^), with infrared thermography further confirming higher surface temperatures relative to neat epoxy under heating [91]. Similarly, Jiao et al. prepared nacre‐like papers based on cellulose nano fibers (CNF) and MXene using vacuum‐assisted assembly, where the in‐plane thermal conductivity rose from 2.58 W m^−1^ K^−1^ in pure CNF films to 14.93 W m^−1^ K^−1^ at 60 wt.% MXene. Their infrared imaging on LED‐heated films revealed ∼ 40°C higher surface temperatures compared with CNF, underscoring the effectiveness of MXene nanosheets in enhancing heat transport in polymer‐based systems [92].

In addition to the intrinsic effect of MXene, the unique morphological architecture of the yarns plays a decisive role in boosting heat transfer. Unlike conventional electrospun mats composed of randomly oriented fibers, the compact and tightly intertwined configuration of nanofibers within the yarns promotes denser fiber‐fiber junctions and the formation of continuous heat‐transfer pathways [93]. This structural arrangement is particularly effective in improving through‐plane thermal conduction, which is one of the main challenges in electrospun systems, without the need for additional processing steps or post‐treatment. Moreover, textile structures based on electrospun yarns generally exhibit superior mechanical strength compared to electrospun membranes [94,95]. Owing to this synergistic combination of structural integrity, scalability, and multifunctional performance, PAN‐MXene yarns represent a promising candidate for thermoregulating textiles, wearable electronics, and multifunctional sensing systems, where efficient heat dissipation and mechanical durability are simultaneously required.

### 2.4 Surface Potential and Energy Harvesting of the Nanofibers

The surface potential of PAN and PAN‐MXene nanofibers was analyzed using KPFM, which enables nanoscale mapping of the contact potential difference (CPD) between a conductive tip and the sample. When the tip and sample have different work functions, an electrostatic force arises, which is compensated by applying a DC bias equal to the CPD, nullifying this force. This allows quantitative visualization of local surface potential, charge distribution, and work‐function variations with nanometer resolution [96,97]. Since surface potential reflects the material's tendency to donate or accept electrons, it can be used to assess its position on the triboelectric series, where materials with higher surface potential are typically tribo‐positive, while those with lower potential are tribo‐negative [98,99].

**FIGURE 5:**
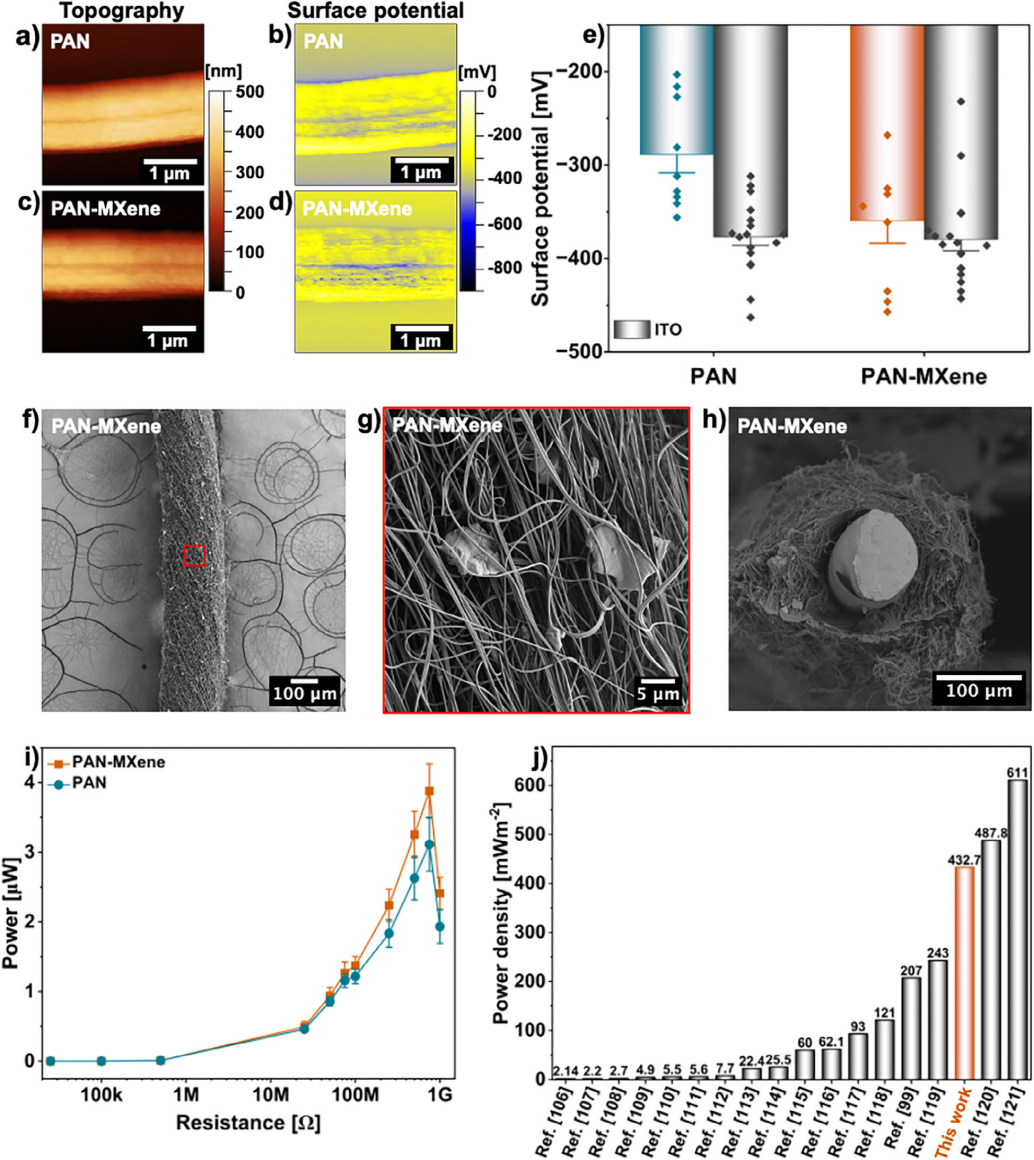
The topography and surface potential maps of (a,b) PAN and (c,d) PAN‐MXene fibers obtained from KPFM measurement; (e) Column chart presenting the average surface potential calculated for the nanofibers and their respective ITO glass slides. Error bars indicate SE. For nanofibers, individual data points correspond to independent measurements (*n* = 9). For ITO, the plotted points represent values extracted from two scan lines per independent sample (total = 18). (f–h) SEM micrographs of the surface morphology and cross‐section of PAN‐MXene yarn coated on copper wire. (i) Power output of PAN and PAN‐MXene yarns coated on copper wire under various external resistances. Error bars denote SE (*n* = 3). (j) Comparison of the power density of PAN‐MXene yarns in this work with other yarn‐based TENGs reported in the literature.

Figure 5a–d demonstrates the representative AFM topography and the corresponding surface potential maps of the individual PAN and PAN‐MXene nanofibers on ITO glass substrates. As summarized in Figure 5e, pristine PAN nanofibers exhibited an average surface potential of −288.7 ± 19.7 mV, placing them in the tribo‐negative region of the triboelectric series, which is consistent with previous reports. Incorporation of MXene further decreased the surface potential to −359.4 ± 24.1 mV, indicating a higher work function and stronger tribo‐negative character for the composite nanofibers. This shift can be attributed to several factors. MXene surfaces are generally terminated with electronegative groups (‐O, ‐F, ‐OH), which introduce localized dipoles and act as charge‐trapping sites that stabilize negative charges [100]. In addition, the lower work function of MXene relative to PAN promotes interfacial electron transfer until Fermi‐level equilibrium is reached, leading to electron accumulation on the PAN side. Hydrogen bonding between the nitrile groups of PAN and ‐OH terminations on MXene further enhances charge localization near the interface, resulting in an overall reduction in surface potential [50,101]. A similar effect has been reported in other MXene‐polymer systems. For instance, incorporating 16.7 wt.% Ti_3_C_2_T_x_ MXene into PLLA fibers markedly reduced their surface potential from −9.3 to −415.7 mV [61]. Similarly, Liu et al. found that adding MXene to PDMS films lowered the surface potential from 0.12 to −3.94 V, reflecting a more negative and electron‐rich surface [102]. It should be noted that pristine MXenes and their composites, particularly Ti_3_C_2_T_x_, have also exhibited tribo‐positive characteristics in some studies [103]. Such discrepancies arise from two main factors: the relative nature of surface‐potential measurements and variations in synthesis conditions. In KPFM, the measured potential depends on the work‐function difference between MXene and the counter material (AFM tip or contact partner); hence, variations in probe composition or calibration can alter the apparent polarity. In addition, the triboelectric properties of MXene are highly sensitive to synthesis parameters, including the etching method, choice of etchant, intercalating agents, and post‐treatment steps. Shi et al. showed that different etching processes yield varying proportions of surface terminations, which play a decisive role in defining the triboelectric response of MXene. Furthermore, parameters such as the intercalating agents and washing duration substantially influence the surface functionality and charge state, leading to differences in the observed triboelectric performance [104].

Given the noticeable reduction in surface potential and enhanced tribo‐negative characteristics induced by MXene incorporation, the relationship between surface potential and triboelectric performance was further examined by fabricating yarn‐based triboelectric nanogenerators (TENGs). The yarn configuration was chosen for its compatibility with textile integration and because PAN‐MXene nanofibers had already demonstrated excellent thermal performance in this form. The composite and pristine PAN yarns were produced by electrospinning directly onto copper wire following the same coating procedure used for samples coated on resistive wire, see Video S2. As shown in Figure 5f–h and Figure S5a–c, the fabricated yarns exhibited uniform coatings with partial fiber alignment along the wire axis.  The overall yarn diameters, including the core wire, were 226 ± 6 µm for PAN and 238 ± 6 µm for PAN‐MXene, and the average fiber diameters were 344 ± 5 and 345 ± 4 nm, respectively (Figure S5d,e), indicating comparable morphology and contact area.

To evaluate their triboelectric performance, the yarns were used as the tribo‐negative component and repeatedly tapped against a tribo‐positive nylon textile, with the copper core serving as the electrode. Figure S6a, represents the working principle of the triboelectric setup. The generated current and power output of the yarns across various resistances are shown in Figure S6b and Figure 5i, respectively. The PAN‐MXene yarns exhibited a maximum power output of 3.88 ± 0.39 µW at 750 MΩ, approximately 25% higher than that of the PAN yarns (3.11 ± 0.38 µW) under identical conditions. Since both samples had similar dimensions and morphology, the observed improvement is attributed to the more negative surface potential of PAN‐MXene fibers, which enhances electron attraction and charge retention during contact‐separation cycles.

A similar correlation between surface potential and triboelectric output has been reported in other polymer composites. Sukumaran et al. found that adding reduced graphene oxide (rGO) to PVDF fibers decreased the surface potential from −0.31 to −0.55 V, due to higher surface electronegativity and charge‐trapping capability, leading to an increase in power density from 0.57 to 3.37 mW cm^−2^ [30]. Similarly, Busolo et al. demonstrated that controlling electrospinning polarity modulated the surface potential of polymethyl methacrylate (PMMA) fibers from 0.6 to 1.2 V, resulting in a fivefold rise in triboelectric output from 46.6 to 234.4 nW at a load resistance of 80 MΩ [105]. These findings consistently indicate that tuning the surface potential, either toward higher or lower values depending on triboelectric polarity, directly governs charge transfer and energy‐harvesting efficiency. However, it should be noted that absolute comparisons of triboelectric outputs across different studies are not straightforward, as triboelectric outputs are highly dependent on device architecture, testing conditions, and normalization methods. Variations in contact area, applied force, frequency, or humidity can lead to substantial discrepancies between reported values. To provide a performance benchmark for the PAN‐MXene triboelectric yarn, the peak power density was selected as the most commonly reported performance indicator in previous studies, although it does not fully capture all device‐dependent effects. Our yarns exhibited a maximum power density of 432.7 mW m^−2^ at 750 MΩ load resistance, corresponding to a contact area of 8,97 mm^2^. As illustrated in Figure 5j, this output ranks among the highest reported for electrospun yarn‐based TENGs in the literature [99, 106, 107, 108, 109, 110, 111, 112, 113, 114, 115, 116, 117, 118, 119, 120, 121]. Moreover, the TENGs exhibited stable performance over 12 000 operation cycles, confirming their mechanical robustness. As shown in Figure S7, the morphology of the yarns after the triboelectric measurements revealed no signs of delamination or cracking, and preserved their fibrous structure, indicating strong adhesion and resistance to surface degradation under repeated contact cycles. Minor wear marks were observed at the contact regions with the counter material, where the nanofibers were partially deformed into a film‐like structure. Overall, the PAN‐MXene yarn‐based TENGs demonstrate excellent durability and are highly promising for integration into smart textiles as flexible, self‐powered sensing and energy‐harvesting components.

### 2.5 Application of Yarns for Tactile Sensing

**FIGURE 6:**
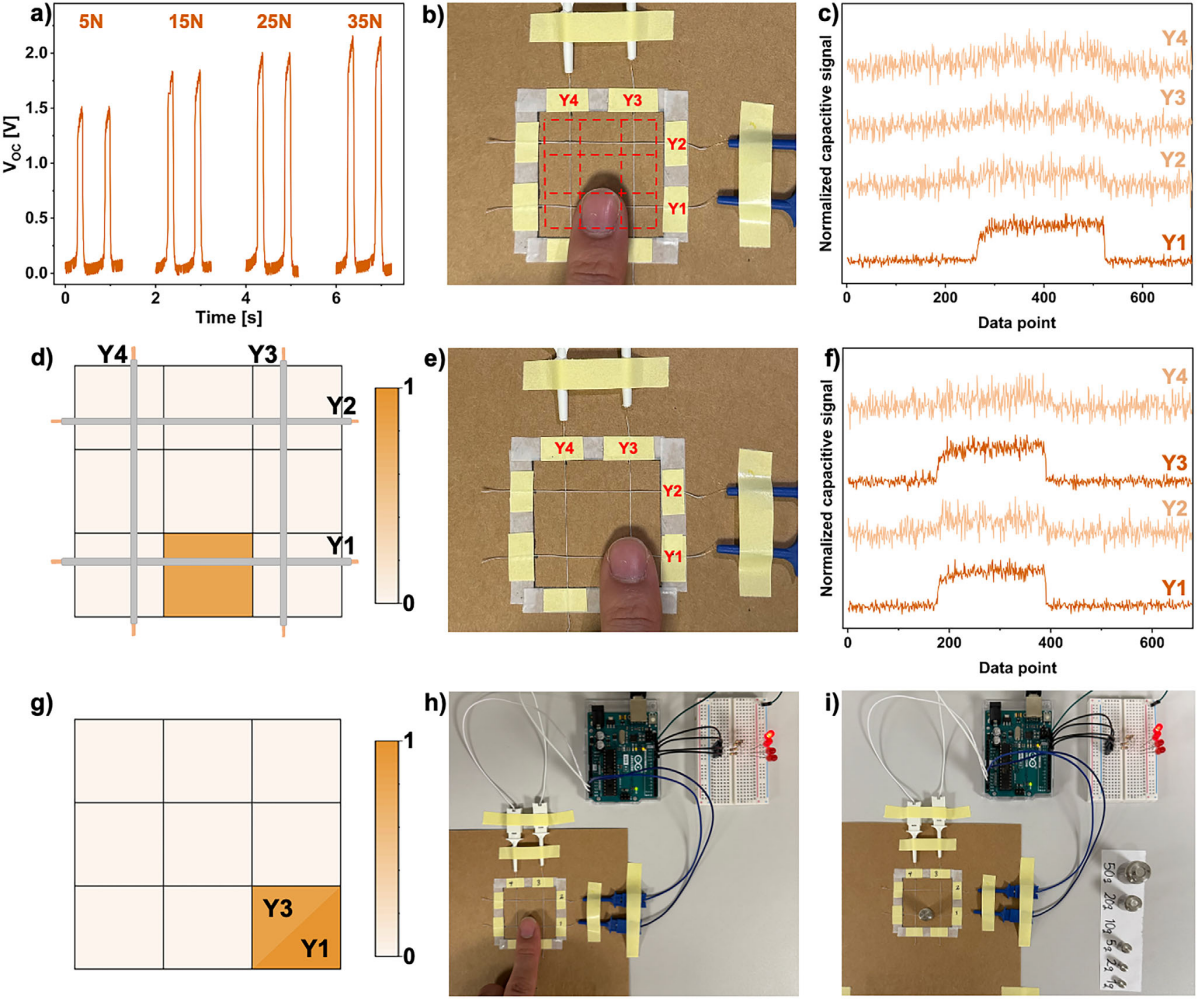
(a) V_OC_ signals demonstrating the force‐sensing sensitivity of the PAN‐MXene yarn. (b) Photograph of the 3 × 3 pixel tactile‐sensing array under finger touch, and (c,d) the corresponding output capacitive signals and the detected touch points visualized as color maps. (e) Photograph of the array touched at a different position, and (f,g) the respective output signals and the color map of the touch location. Photographs showing the tactile‐sensing array used to activate LEDs through (h) finger touch and (i) applying different weights.

Beyond their energy‐harvesting performance, the PAN‐MXene yarns also demonstrated effective force‐sensing functionality. As shown in Figure 6a, the open‐circuit voltage (V_OC_) of the composite yarns increased almost linearly with the applied tapping force in the range of 5–35 N, confirming their effective force‐sensing capability. To further explore their sensing potential, the composite yarns were integrated into an active tactile‐sensing array capable of quantitatively recording pressure stimuli and spatially mapping touch positions. As shown in Figure 6b, a 3 × 3 pixel sensing array was constructed by arranging two yarns horizontally across two vertically aligned ones. This simple configuration can be easily integrated into textile structures. Each yarn functions as an independent electrode, and when the array is touched, variations in capacitance are detected as electrical signals, exhibiting instantaneous response and recovery, as shown in Video S3. These signals are continuously recorded and processed in real time using a multichannel data acquisition system connected to a preprogrammed microcontroller, which identifies both the location and magnitude of the applied force. For instance, when a specific region containing only yarn Y1 was touched (Figure 6b), a signal appeared solely on the Y1 channel (Figure 6c). In contrast, touching the intersection of Y1 and Y3 (Figure 6e) generated simultaneous signals on both corresponding channels (Figure 6f). The acquired data were subsequently used to generate heat maps representing the activated pixel locations (Figure 5d,g).

The yarns were further employed as an interface for human‐machine interactions. As illustrated in Figure 6h and Video S4, the microcontroller was programmed to translate the output signal from each yarn into a command to activate a corresponding LED. Each yarn (Y1‐Y4) was linked to an individual LED that illuminated when the respective yarn was touched, demonstrating a clear and selective signal response. Building on the previously confirmed force‐sensing capability, this functionality was also tested in a practical scenario. Because the recorded electrical signal depends on the applied force, the program was adjusted to respond only when the input exceeded a defined threshold. Below this threshold, the sensor remained inactive, whereas forces above it triggered LED activation as an indicator. Using standard weights from 1 to 50 g (Figure 6i), the setup selectively responded to loads exceeding 10, 20, and 50 g (Videos S5–S7), corresponding to a minimum detectable force of ∼ 0.1 N (approximate applied force by the 10 g weight). These results highlight the strong potential of PAN‐MXene yarns for tactile sensing and human–machine interface applications. Moreover, their inherent flexibility and knittability make them highly suitable for integration into smart textiles capable of detecting touch and human activity in next‐generation wearable systems.

While this study focused on evaluating the multifunctional performance of the PAN‐MXene composite fibers and yarns, it is also important to acknowledge the issue of environmental stability, particularly given the Ti_3_C_2_T_x_ MXenes susceptibility to oxidation, especially under conditions involving elevated temperature or mechanical stress, which can adversely affect their long‐term thermal and electrical properties. To address this problem, previous studies have shown that embedding MXene within a polymer matrix can significantly suppress oxidation by limiting exposure to oxygen and moisture [122, 123, 124]. In our composites, the partial encapsulation of MXene flakes by the PAN matrix is expected to help preserve their structural integrity and functional performance during thermal or mechanical use. Although long‐term environmental stability testing was beyond the scope of this work, it is worth noting that all thermal and electrical measurements were conducted on samples stored under ambient conditions for several months, during which they maintained stable and high performance. Moreover, most of the reported studies on MXene oxidation involve accelerated degradation under extreme conditions (e.g., prolonged exposure to temperatures above 100°C or high humidity), which are not representative of the operating conditions anticipated for the materials presented here [125,126]. Nevertheless, the long‐term effects of MXene oxidation on the performance of PAN‐MXene composites remain an important consideration and warrant further investigation in future work.

## 3 Conclusion

Electrospun PAN‐MXene nanofibers and yarns were fabricated as unique multifunctional composites combining enhanced heat dissipation, photothermal response, triboelectric performance, and tactile sensing. Despite the high loading of Ti_3_C_2_T_x_ MXene within the PAN matrix, the resulting composite nanofibers retained excellent flexibility. DSC analysis revealed a clear shift of the PAN cyclization peak toward higher temperature, indicating improved thermal stability. The composite nanofibers also exhibited strong photothermal conversion, with their temperature rapidly rising to 60°C under IR illumination. More importantly, the incorporation of MXene established continuous thermal pathways that enhanced phonon transport across multiple length scales. SThM confirmed superior nanoscale heat conduction in individual nanofibers, while IR thermography showed a ∼ 22°C higher surface temperature for composite yarns compared to pristine PAN, evidencing enhancement in thermal conductivity. Furthermore, KPFM demonstrated reduced surface potential and stronger tribo‐negative behavior for composite nanofibers, which increased the energy harvesting performance of PAN‐MXene yarns by 25%, with a peak power density of 432.7 mW m^−2^. The yarns also displayed reliable force‐sensing performance, capable of detecting forces down to 0.1 N, highlighting their potential for tactile and wearable applications. Collectively, these results establish electrospinning as a scalable route to produce lightweight, flexible, and thermally efficient PAN‐MXene composites, offering a versatile platform for heat management, energy harvesting, and sensing in next‐generation electronic and wearable systems.

## 4 Experimental Section

### 4.1 Sample Preparation

#### 4.1.1 Electrospinning

Polyacrylonitrile (PAN, Mv = 150 000; Sigma–Aldrich, UK) was dried at 30°C for 4 h in a drying oven (POL‐ECO Aparatura, Poland) prior to solution preparation. A 9 wt.% PAN solution in dimethylformamide (DMF) was prepared by stirring at 200 rpm on a heating plate (IKA, Germany) for 18 h. For PAN‐MXene solutions, multilayer Ti_3_C_2_T_x_ MXene nanoflakes (Nanochemazone, Canada, CAS NO: 12363‐89‐2), with a reported thickness of 100–200 nm, were first dispersed in DMF using an ultrasonic bath (Sonorex Bandelin, Germany) for 1 h in an ice bath. PAN was then added to the dispersion and stirred for 18 h at 200 rpm. The MXene content was adjusted to 50 wt.% relative to the PAN content. Prior to electrospinning, the MXene‐containing solution was further ultrasonicated for 1 h to ensure uniform dispersion. Electrospinning was performed using a machine from IME Technologies (The Netherlands) equipped with a climate control chamber set to a temperature (T) of 25°C and 40% relative humidity (RH). The rest of the electrospinning parameters were as follows: applied voltage of 15 kV, flow rate of 1 mL h^−1^, nozzle to collector distance of 10 cm, and a 21‐gauge stainless steel nozzle.

Coated yarns were produced using an electrospinning setup equipped with a yarn‐spinning module (TechNOVA, China) on either a resistive wire (RD 100/02 Block, Germany) or a copper wire (diameter of 100 µm) serving as the core. The yarn electrospinning was carried out under controlled environmental conditions, at RH = 30%–37% and T = 25°C. Positive and negative voltages of 14 and 15 kV, respectively, were applied to the nozzles, and the polymer solutions were fed at a rate of 0.01 mL min^−1^. The rotation speeds of the vortex collector and collecting mandrel were 300 and 10 rpm, respectively. For yarns coated on the copper wire, the nozzle‐to‐vortex collector distances were set to x = 11–12 cm and y = 9–12 cm for the positive nozzle, and x = 13 cm and y = 9–10 cm for the negative nozzle. In the case of yarns coated on the resistive wire, the corresponding distances were x = 10–12 cm and y = 10–12 cm for the positive nozzle, and x = 13 cm and y = 10–11 cm for the negative nozzle. The schematic in Figure S4a, illustrates how the x and y distances are defined and measured relative to the collector and nozzle positions.

### 4.2 SEM, EDS, and FTIR

The morphology of the produced fibers was examined using scanning electron microscopy (SEM, Merlin Gemini II, ZEISS, Germany). Prior to imaging, the samples were coated (sputter coater Qi50RS, Quorum Technologies, UK) with an 8 nm‐thick Au layer. SEM imaging was performed at an accelerating voltage of 2.5 kV and working distance of 5.2–5.7 mm, using a secondary electron (SE) detector. The morphology of the yarns was investigated under similar conditions, using a working distance of 6–6.3 mm. Average fiber diameters (D) were calculated from measurements of 100 randomly selected fibers using ImageJ software (v. 1.54 g, USA) based on the acquired SEM images. To evaluate the morphology and lateral size distribution of the MXene nanoflakes, SEM imaging was performed on Ti_3_C_2_T_x_ MXene dispersed in DMF after 1 h of ice‐bath ultrasonication. The suspension was drop‐cast onto Si wafers pre‐coated with an 8 nm Au layer and dried under ambient conditions. Imaging was conducted at an accelerating voltage of 2.5 kV, with a 3.4 mm working distance, using an SE detector. Flake dimensions were determined by measuring Feret's diameter and equivalent circular diameter (ECD) for 100 randomly selected MXene flakes using ImageJ, and the statistical analysis was performed in OriginPro (2025b, USA). To analyze the distribution of MXene nanoflakes, elemental mapping was conducted via energy‐dispersive X‐ray spectroscopy (EDS, Bruker, Germany). For this purpose, the samples were coated with a ∼ 15 nm‐thick carbon layer using a carbon evaporator (K950, Emitech (Quorum Technologies), UK), and EDS mapping was carried out over 300 s at an accelerating voltage of 15 kV, a current of 1.1 nA, and a working distance of 7.2 mm, using a backscattered electron detector.

The chemical structure of the electrospun samples was analyzed using attenuated total reflectance‐Fourier transform infrared spectroscopy (ATR‐FTIR, Nicolet iS 5, Thermo Fisher Scientific, USA) equipped with a diamond crystal. FTIR measurements were conducted on the as‐spun nanofibers as well as on the samples after DSC measurements. The spectra were obtained by averaging 64 scans collected over the 400–4000 cm^−1^ range at a resolution of 4 cm^−1^. Baseline correction and spectral normalization were performed in OriginPro (2025b, USA) using the Peak Analyzer and Normalize functions, respectively.

### 4.3 Mechanical Test

The mechanical properties of the electrospun nanofibers were evaluated using a tensile testing machine (Kammrath & Weiss, Dortmund, Germany) equipped with a 20 N load cell. Samples were prepared in the form of mats by electrospinning for 1 h and subsequently cut into 12 × 7 mm rectangles. To ensure stable gripping and avoid slippage during testing, the samples were secured to the machine's clamps using double‐sided adhesive tape. Tensile tests were performed at an extension rate of 25 µms^−1^ under ambient conditions (T = 21°C–22°C, and RH = 18%–33%). The average thickness of mats was determined from three separate measurements using a stationary thickness gauge (TMG‐1‐T, CHECKLINE, USA), following the ISO‐5084 standard for textile materials, see Table S2. stress values were calculated by dividing the applied force by the initial cross‐sectional area of each sample. The average maximum stress (𝜎_max_), strain at maximum stress (ε_max_), and toughness (W) were obtained from three separate measurements (Figure S8) using the Integrate function in OriginPro (2025b, USA). The morphology of the nanofibers after the mechanical test was analyzed by SEM at an accelerating voltage of 2.5 kV and a working distance of 5.4–7.1 mm.

### 4.4 DSC and Photothermal Conversion

Thermal characterization of the nanofibers was performed by Differential Scanning Calorimetry (DSC, Model 3, Mettler Toledo, USA) over a temperature range of 25°C–400°C, with a heating rate of 10°C min^−1^. Measurements were conducted under an Ar atmosphere, with the electrospun samples placed in Al crucibles. Average values of exothermic enthalpy and characteristic transition temperatures were determined using STARe software (Mettler Toledo, Switzerland), based on the first heating cycle from three separate measurements per sample, see Figure S8.

To assess the photothermal conversion efficiency of the nanofibers, electrospun mats were irradiated using an infrared (IR) lamp with a power output of 100 W, while their surface temperatures were simultaneously recorded with a thermal camera (FLIR T560, USA). The mats were prepared by electrospinning for 4 h and then cut into 3 × 3 cm squares. For measurement, the samples were vertically suspended on a low‐absorbing plastic stand using small strips of tape to ensure uniform IR exposure and minimize conductive heat loss. A standard tape (Super 33+, Scotch, USA) with a known emissivity of 0.96 was placed next to the samples to provide a reliable temperature reference during analysis. The distance from the IR lamp and thermal camera to the samples were set at 15 and 20 cm, respectively. After 10 min of IR exposure (allowing the samples to reach thermal equilibrium), the lamp was switched off, and the mats were allowed to cool to ambient temperature. All measurements were conducted under similar conditions, within an ambient temperature of 22°C–24°C, and RH = 38%–39%. Average surface temperatures of both the mats and the reference tape were determined using the average box function in FLIR Tools software, based on three separate measurements for each sample.

### 4.5 Scanning Thermal Microscopy

Thermal analysis of individual PAN and PAN‐MXene nanofibers was conducted using an atomic force microscope (AFM, CoreAFM, Nanosurf, Switzerland) with a specialized scanning thermal microscopy module (SThM, VertiSense, AppNano, USA). A VTP‐200 thermal probe (VertiSense, AppNano, USA) with a silicon cantilever was employed for the measurements. The cantilever tip was heated via laser and maintained at a temperature 50°C above room temperature prior to contact with the sample, ensuring a consistent temperature gradient between the tip and the nanofibers. Samples were prepared by shortly electrospinning onto the ITO‐coated glass slides (Ossila, UK), which were then securely mounted onto a metallic stage using silver paste to provide an effective thermal pathway for heat dissipation. Because the size mismatch, complex topography, and insufficient substrate attachment at PAN/MXene junctions resulted in unstable tip‐sample contact and non‐reproducible signals, SThM measurements were performed only on fiber segments. Measurements were performed in contact mode with a scan rate of 2 s per line, a resolution of 256 pixels per line, and a contact force of 20 nN. To eliminate any potential differences in thermal transport arising from probe differences, the same thermal probe was used across all measurements, and all samples were affixed to the same metallic stage. Measurements were carried out under ambient conditions (T = 22.4°C–25.5°C, and RH = 26%–44%), with three scans taken for each sample type at randomly selected locations. The ITO substrate served as a thermal reference to rule out effects from potential fluctuations in room temperature or substrate variability. All AFM data were processed using Gwyddion (v2.56, gwyddion.net) and OriginPro (2025b, USA). For localized spectroscopy‐mode measurements, a procedure adapted from our previous work [35] was followed. In brief, the cantilever's tip was heated to 25°C above ambient temperature, and measurements were performed along 25 randomly selected scan lines on each sample. At each measurement point, the tip approached the sample over 0.5 s, made contact, and remained stationary for 2 s before retracting over another 0.5 s to a distance of 3 µm, followed by an additional 2 s pause after retraction. A stop‐by force of 75 nN was applied during the contact phase. The voltage signal recorded while the tip was paused in contact with the nanofiber surface was used to calculate the average tip temperature. All other experimental settings were kept identical to those used during contact‐mode scanning, and ambient conditions were T = 22°C–24°C, and RH = 40%–52%.

### 4.6 IR Thermography on Coating Yarns

A similar method to our previous study was employed to assess the heat conduction performance of the coating yarns [64]. Briefly, the yarns were heated by applying voltage to the resistive wires, and their surface temperatures were recorded using a thermal camera (FLIR T560, USA) after reaching thermal equilibrium. To ensure consistent heating across samples, a standard tape (Super 33+, Scotch, USA) with a constant emissivity of 0.96 was attached to each wire as a reference. Average surface temperatures of both the yarns and the reference tape were determined using the line averaging function in FLIR Tools software, based on five separate measurements. All measurements were conducted under ambient conditions (T = 21°C, and RH = 23%–25%).

### 4.7 Kelvin Probe Force Microscopy

Kelvin probe force microscopy (KPFM) was performed using a CoreAFM system (Nanosurf, Switzerland) with a conductive HQ:NSC18/Pt probe (MikroMasch, Bulgaria), which has a spring constant of 2.8 Nm^−1^ and a resonance frequency of 75 kHz. Sample preparation followed the same procedure as for the SThM analysis, with nanofibers electrospun onto ITO‐coated glass slides. To minimize variations in the background's surface potential, the ITO substrates were mounted on a metallic stage using silver paste. The measurements were performed in the dynamic mode, and both the topography and surface potential data were collected simultaneously. Surface potential values were determined by averaging scan lines taken from the uppermost part of nine different nanofibers, measured across different scan regions. The measurements on ITO substrates served as reference controls for the KPFM signal, with two scan lines averaged per region. All measurements were conducted under ambient condictiones (T = 23°C–24°C and RH = 13%–17%). Data analysis was performed using Gwyddion (v2.56, gwyddion.net) and OriginPro (2025b, USA).

### 4.8 Energy‐Harvesting and sensing Characterization

The triboelectric energy‐harvesting performance of PAN and PAN‐MXene nanofibers, coated as yarns on copper wire, was evaluated using a custom‐built measurement setup operating in single‐electrode mode. Measurements were performed in a contact‐separation configuration, in which the triboelectric‐negative yarns were cyclically tapped against triboelectric‐positive nylon fabric using a linear motor (LinMot P04, USA). The yarns were mounted on a polymethyl methacrylate holder, while the nylon fabric with the thickness of 0.860 mm, was cut into circular discs of 24 mm diameter and affixed to a polyethylene terephthalate foam attached to an Al stub with carbon tape. Tapping parameters were set to a frequency of 1.5 Hz, a contact force of 20 N, and a separation distance of 20 mm between the yarns and the nylon fabric. Current output was measured using an electrometer (Keithley 6517B, Cleveland, USA) across external load resistances ranging from 0 to 1GΩ. To stabilize the electrical output, all measurements were recorded after 30 min of continuous tapping. Peak‐to‐peak current values were extracted using the Peak Analyzer function in OriginPro (2025b, USA), and current and power outputs were calculated by averaging data from three independent samples measured on different days (i.e., varying room temperature and relative humidity). The open‐circuit voltage (V_OC_) of the yarns under varying contact forces was measured using the same electrometer (Keithley 6517B). For this measurement, the applied force was adjusted by changing the current supplied to the linear motor. The rest of the tapping parameters were kept the same as those used for measuring current output. The capacitive tactile sensing performance of the composite yarns was evaluated using a programmable microcontroller (Arduino Uno R3, Italy). A Python program was used to extract the real‐time recorded data, which were further analyzed and processed to prepare heat maps using OriginPro (2025b, USA) software. To assess their response to the applied force, a series of standard calibration weights (KERN & Sohn GmbH, Germany) ranging from 1 to 50 g was utilized in force‐responsive touch sensing measurements.

### 4.9 Statistical Analysis

All statistical analyses were performed using OriginPro (2025). FTIR spectra were baseline‐corrected and normalized between 0 and 1, and tactile‐sensing signals were similarly normalized prior to generating heat maps. Quantitative results were reported as mean ± standard error (SE). Sample sizes for each experimental series are provided in the corresponding figure captions or in the Experimental Section, with no fewer than three independent replicates for each experiment. For electrospun mats and yarns, these correspond to distinct samples prepared under identical electrospinning conditions, while for microscopic measurements they refer to separate measurement points or locations on the sample. No statistical hypothesis testing was performed, as the study does not involve comparisons requiring significance analysis. Error bars in the figures represent SE, and individual data points were shown where appropriate to illustrate the measurement variability and reproducibility.

## Conflicts of Interest

The authors declare no conflicts of interest.

## Supporting information




**Supporting File**: adma71984‐sup‐0001‐SuppMat.docx

**Supplementary Video 1**: adma71984‐sup‐0002‐VideoS1.mov

**Supplementary Video 2**: adma71984‐sup‐0003‐VideoS2.mov

**Supplementary Video 3**: adma71984‐sup‐0004‐VideoS3.mov

**Supplementary Video 4**: adma71984‐sup‐0005‐VideoS4.mov

**Supplementary Video 5**: adma71984‐sup‐0006‐VideoS5.mov

**Supplementary Video 6**: adma71984‐sup‐0007‐VideoS6.mov

**Supplementary Video 7**: adma71984‐sup‐0008‐VideoS7.mov

## Data Availability

The data that support the findings of this study are available from the corresponding author upon reasonable request.
