# Supplementary material for: Scalable and Multifunctional PAN‐MXene Composite Fibers for Thermal Management, Photothermal Conversion, Energy Harvesting, and Sensing for Wearable Applications
Source: Adv Mater. 2025 Dec 30;38(26):e22098. doi: 10.1002/adma.202522098 (PMC13155330; doi:10.1002/adma.202522098)
Supplement: Supplementary file 1 — Supporting File: adma71984‐sup‐0001‐SuppMat.docx [file ADMA-38-e22098-s003.docx]

Supporting Information

**Scalable and Multifunctional PAN-MXene Composite Fibers for Thermal management, Photothermal Conversion, Energy Harvesting, and Sensing for Wearable Applications**

*Ahmadreza Moradi, Piotr K. Szewczyk, and Urszula Stachewicz^*^*

A. Moradi, P. K. Szewczyk, U. Stachewicz

Faculty of Metals Engineering and Industrial Computer Science

AGH University of Krakow

Krakow 30–059, Poland

E-mail: [ustachew@agh.edu.pl](mailto:ustachew@agh.edu.pl)

In this supporting information, we provide complementary data and figures supporting the results discussed in the main manuscript. Figure S1 presents SEM micrographs and lateral size distributions of the multilayer MXene nanosheets. Table S1 summarizes the mechanical properties of electrospun PAN and PAN-MXene mats. Figure S2 shows ATR-FTIR spectra of cyclized PAN and PAN-MXene nanofibers after DSC measurements. Figure S3 provides nanoscale thermal conductivity analyses obtained from SThM, comparing the local temperature distribution on individual PAN and PAN-MXene nanofibers and the ITO background. Figure S4 illustrates the schematic of the yarn electrospinning process and SEM micrographs of the surface morphology and cross-section of the PAN-coated resistive wire, together with the statistical analyses of average yarn and fiber diameters. Figure S5 shows the corresponding SEM micrographs for PAN yarns coated on copper wire, along with the respective diameter distributions. Figure S6 depicts the schematic of the experimental setup used to evaluate the triboelectric energy-harvesting performance of the yarns, accompanied by the measured current output across various external resistances, and representative short-circuit current signals of PAN-MXene yarns and copper wire. Figure S7 shows SEM micrographs of PAN and PAN-MXene triboelectric yarns after 12 000 tapping cycles. Figure S8 presents the stress-strain curves and DSC thermograms of the electrospun nanofibers. Table S2 reports the thicknesses of the electrospun mats used for the mechanical and photothermal measurements, and Figure S9 shows a photograph of the PAN-MXene yarns collected after the yarn electrospinning process.


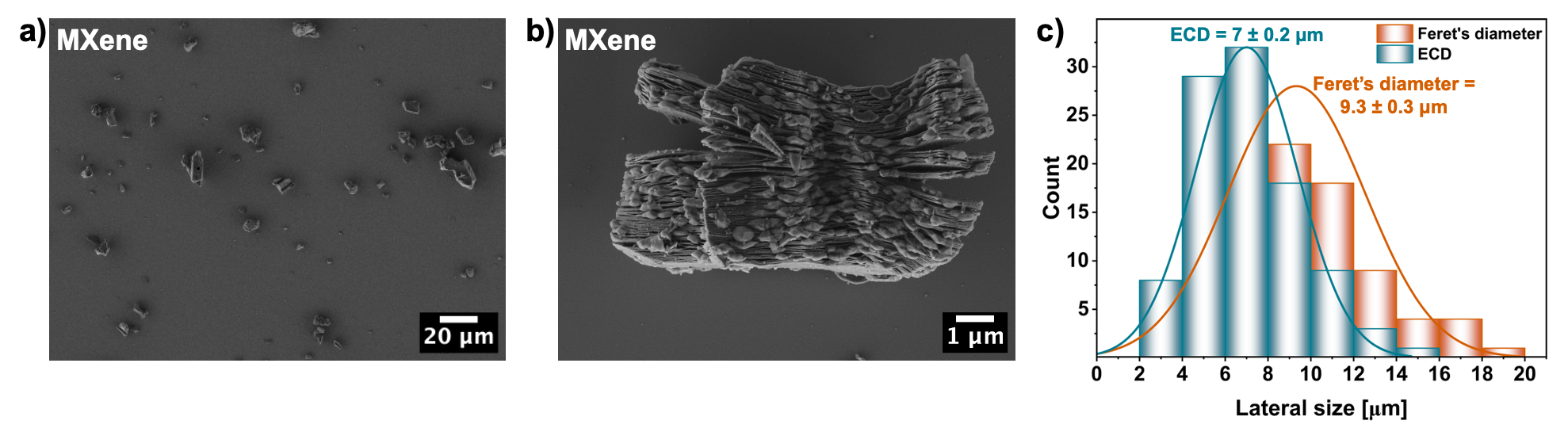


**Figure S1.** a-b) SEM micrographs illustrating the morphology of multilayer MXene nanosheets. c) Lateral size distributions of MXenes obtained from Ferets’ diameter and equivalent circular diameter (ECD) measurements. Data are presented as mean ± SE (n = 100 flakes per sample).

**Table S1**. The summary of the mechanical properties of the electrospun mats. Data are presented as mean ± SE (n = 3).

|  | **PAN** | **PAN-MXene** |
| --- | --- | --- |
| **Maximum stress, σ_max_ [MPa]** | 3.25 ± 0.05 | 2.74 ± 0.02 |
| **Strain at maximum stress, ε_max_ [%]** | 78 ± 2 | 75 ± 2 |
| **Strain at failure, ε_f_ [%]** | 241 ± 19 | 178 ± 11 |
| **Toughness, W [MJm^-3^]** | 3.16 ± 0.08 | 2.54 ± 0.04 |


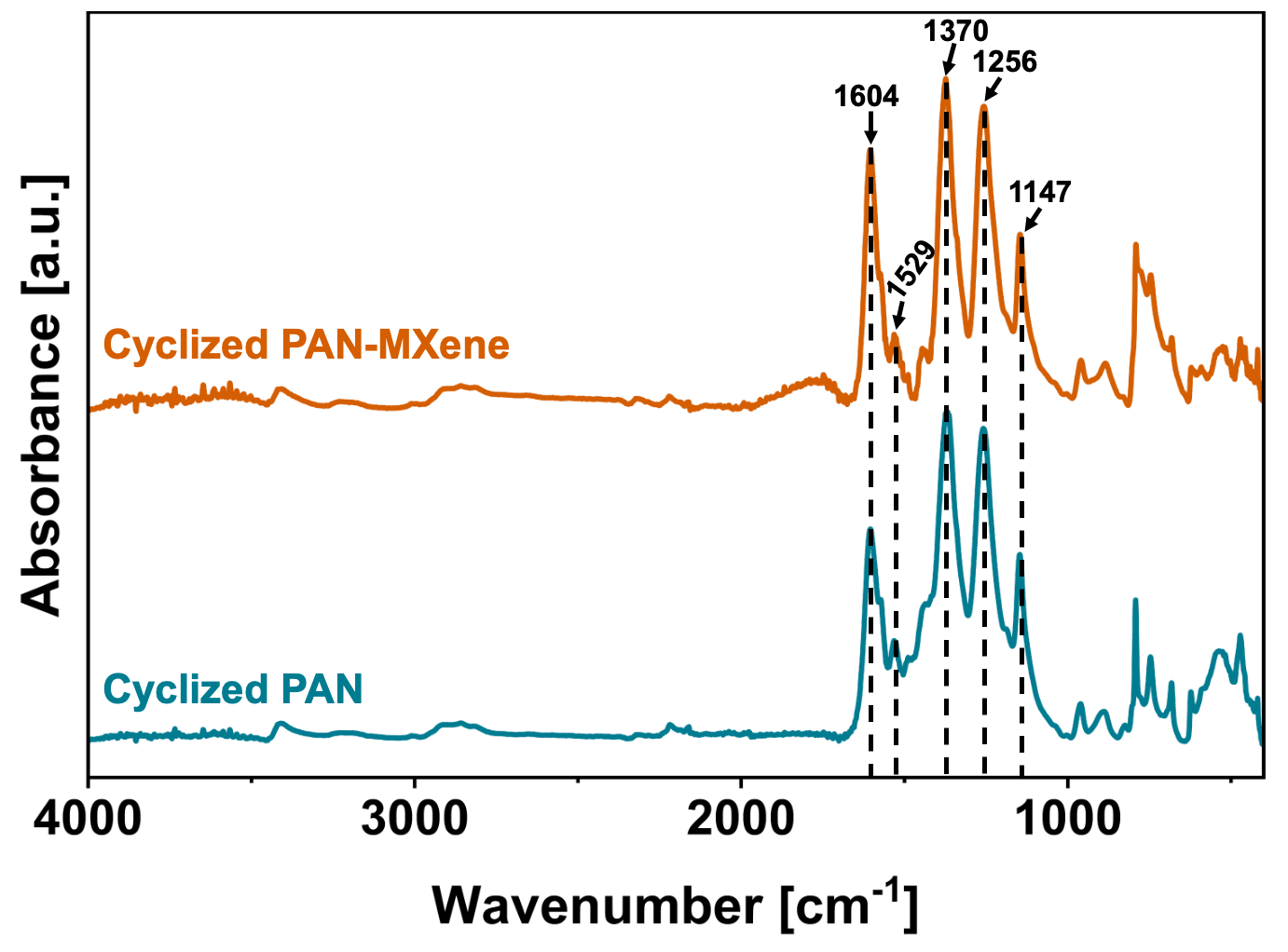


**Figure S2.** ATR-FTIR spectra of cyclized electrospun PAN and PAN-MXene nanofibers after the DSC measurements.


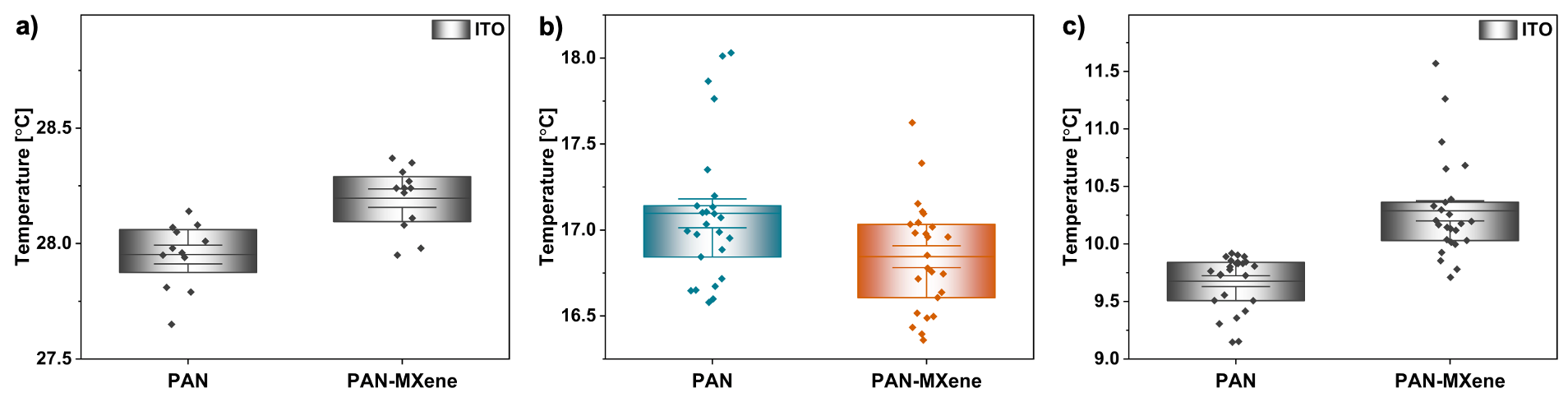


**Figure S3.** a) Box charts showing the average tip temperature on ITO substrates beneath PAN and PAN-MXene nanofibers, obtained from thermal conductivity maps of SThM measurements. (b) Box charts comparing the average tip temperature on individual nanofibers and c) on their corresponding ITO backgrounds, obtained from spectroscopy mode of SThM. Boxes represent the 25 – 75% range; the central line indicates the mean; whiskers denote SE. Data points for the thermal conductivity maps (total = 12) were obtained from four scan lines on ITO within each of the three independent scan regions (n = 3). For the spectroscopy mode, data points correspond to individual measurement positions for each sample type (n = 25).


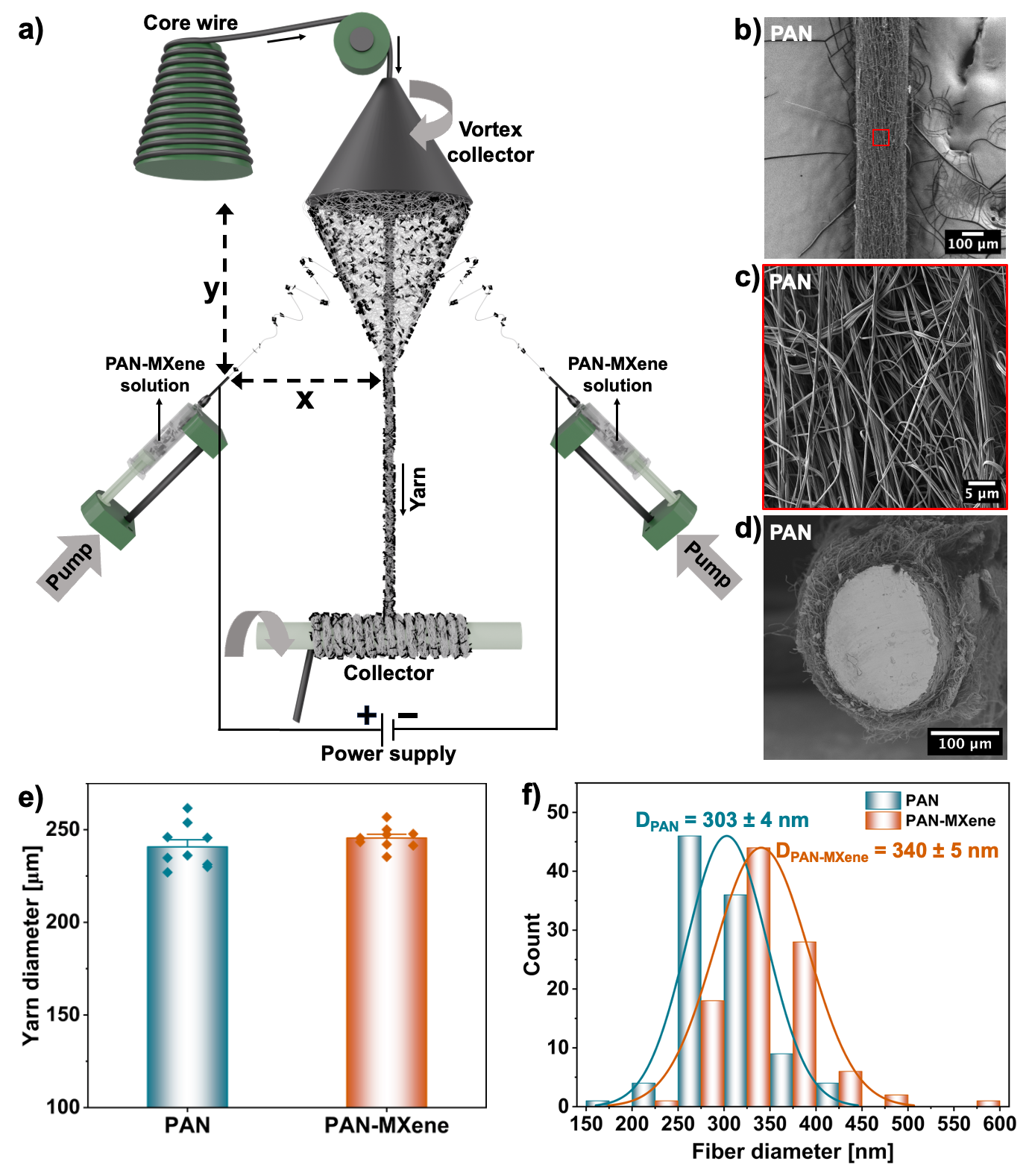


**Figure S4.** a) Schematic illustration of the yarn fabrication process. b-d) SEM micrographs demonstrating the surface morphology and cross-section of electrospun PAN yarns coated on resistive wire. e) Column chart presenting the average yarn diameter. Error bars indicate SE (n = 3). Data points (total = 9) were derived from three measurements for each of the three separate yarns per sample type. f) Corresponding fiber diameter distribution curves with the calculated average fiber diameters. Data are presented as mean ± SE (n = 100 fibers per sample).


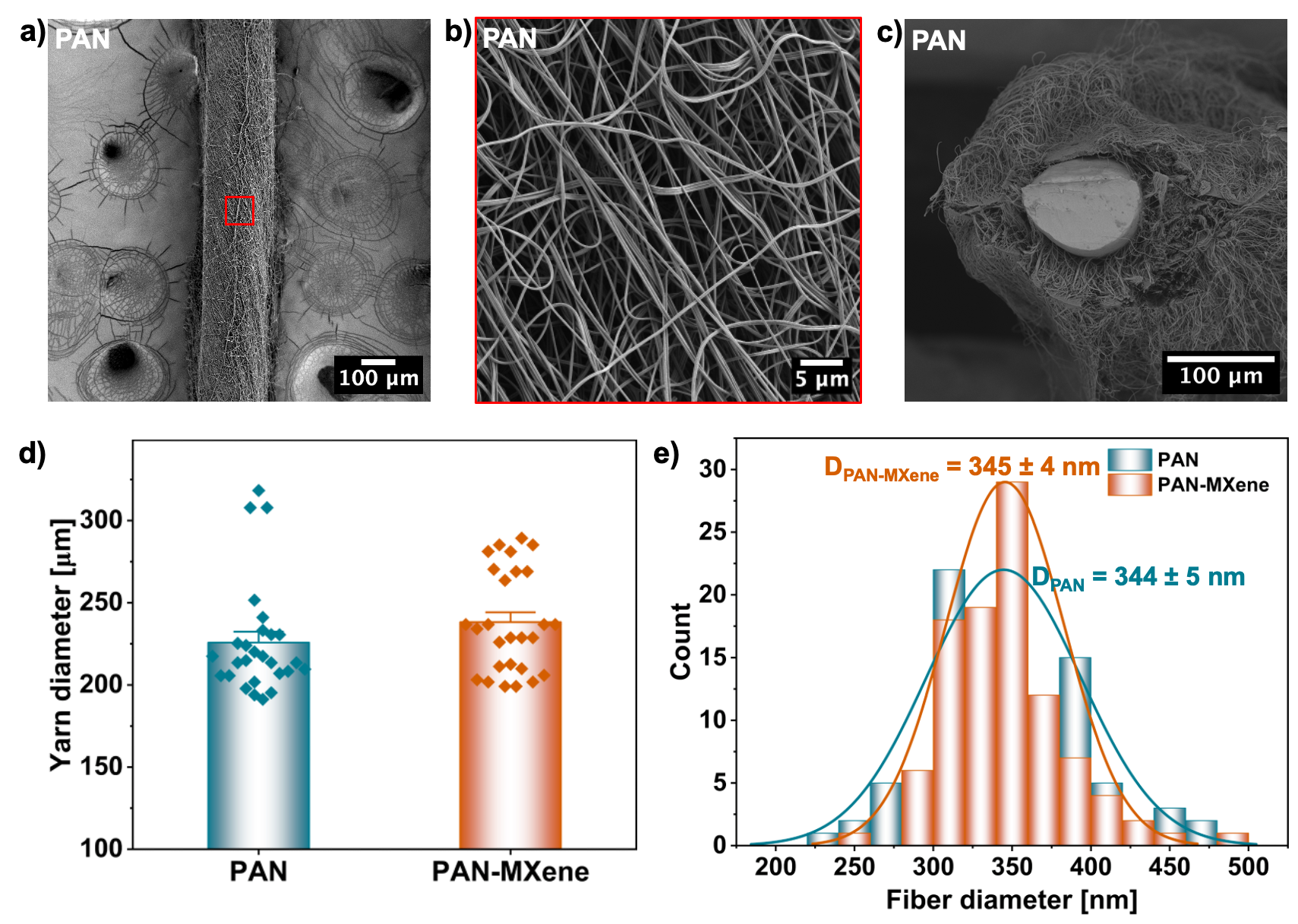


**Figure S5.** a-c) SEM micrographs of the surface morphology and cross-section of PAN-MXene yarn coated on copper wire. d) Column chart presenting the average yarn diameter. Error bars denote SE (n = 9). Data points (total = 27) were obtained from three measurements for each of the nine separate yarns per sample type. f) Corresponding fiber diameter distribution curves with the calculated average fiber diameters. Data are presented as mean ± SE (n = 100 fibers per sample).


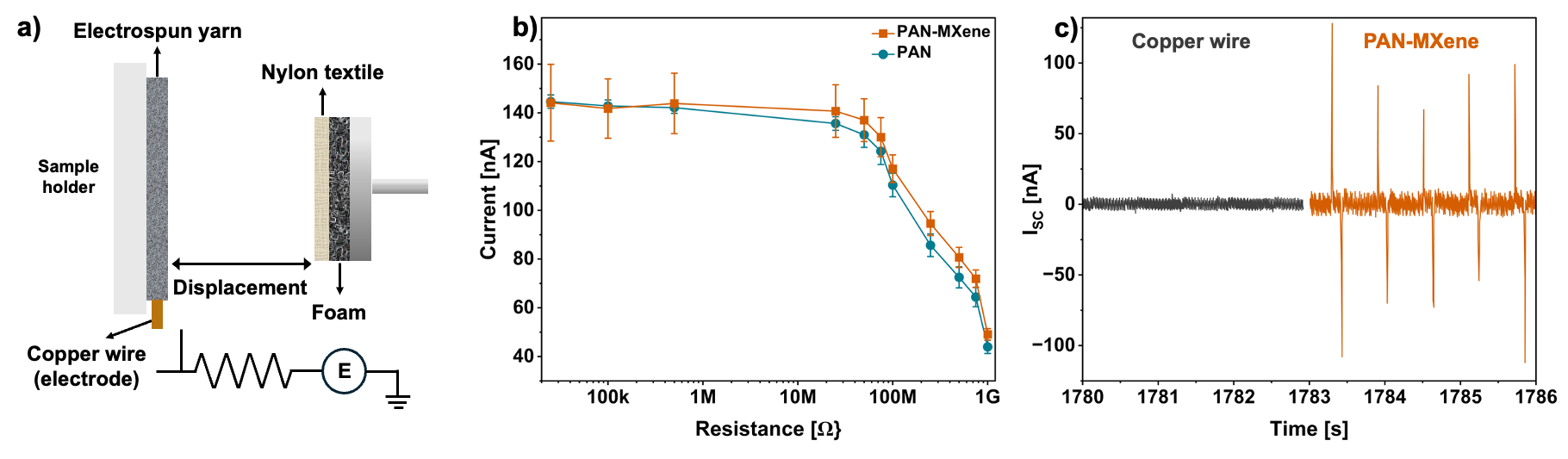
**Figure S6.** a) Schematic representation of the setup used to characterize the energy harvesting performance of the PAN and PAN-MXene yarns. b) Current output of the electrospun yarns measured across several external resistances. Error bars indicate SE (n = 3). c) Examples of measured short-circuit current (I_SC_) signals of the PAN-MXene yarns and copper wire.


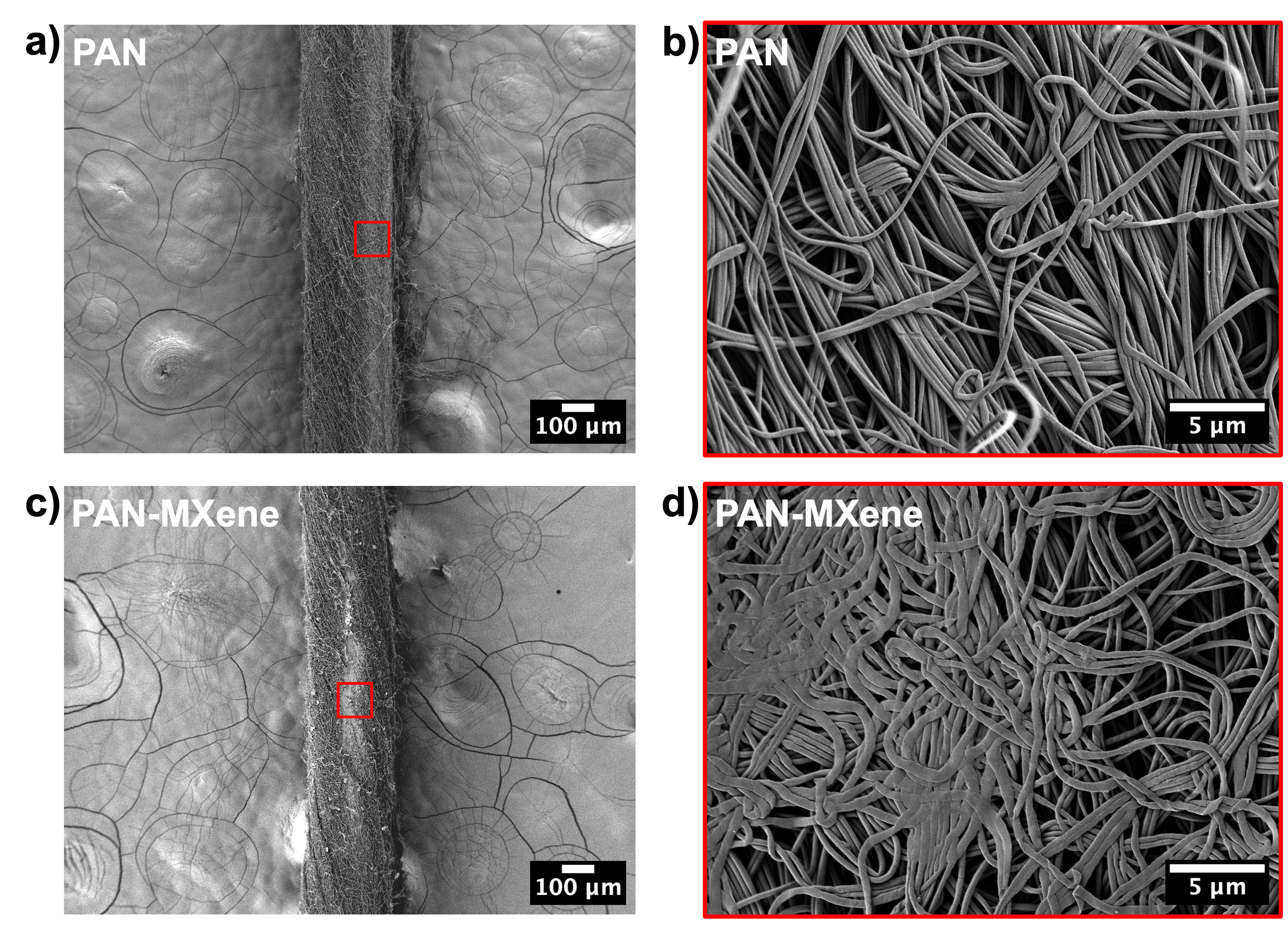


**Figure S7.** SEM micrographs showing the surface morphology of a-b) PAN and c-d) PAN-MXene triboelectric yarns after 12000 tapping cycles.


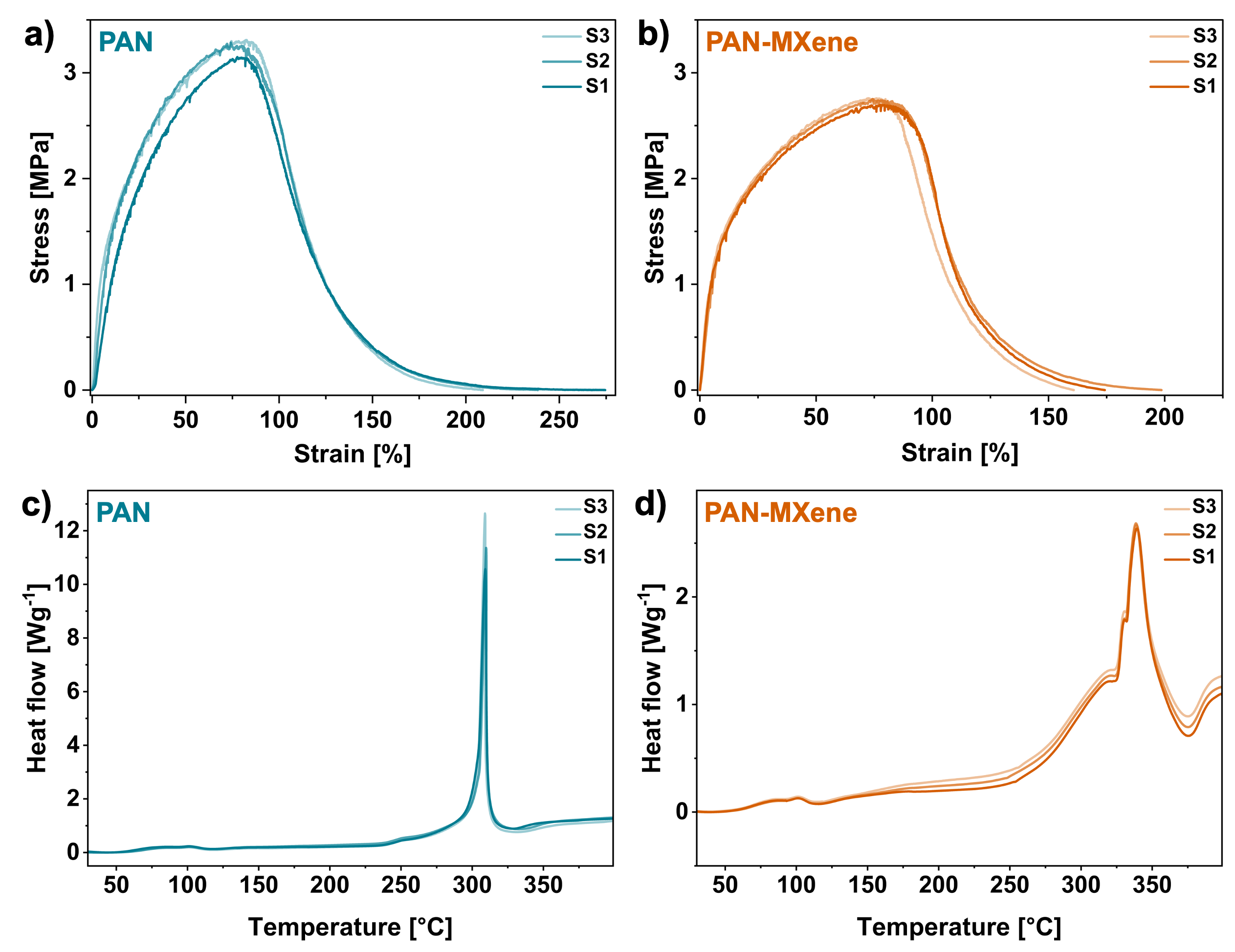


**Figure S8.** Stress-strain curves of the electrospun a) PAN and b) PAN-MXene nanofibers. DSC heating thermograms of c) PAN and d) PAN-MXene nanofibers.

**Table S2.** The thickness of the electrospun mats used for tensile test and photothermal conversion measurements. Data are presented as mean ± SE (n = 3).

|  | **PAN** | **PAN-MXene** |
| --- | --- | --- |
| **The thickness of the electrospun mats for the tensile test [μm]** | 45 ± 3 | 99 ± 5 |
| **The thickness of the electrospun mats for the photothermal measurement [μm]** | 348 ± 6 | 694 ± 5 |


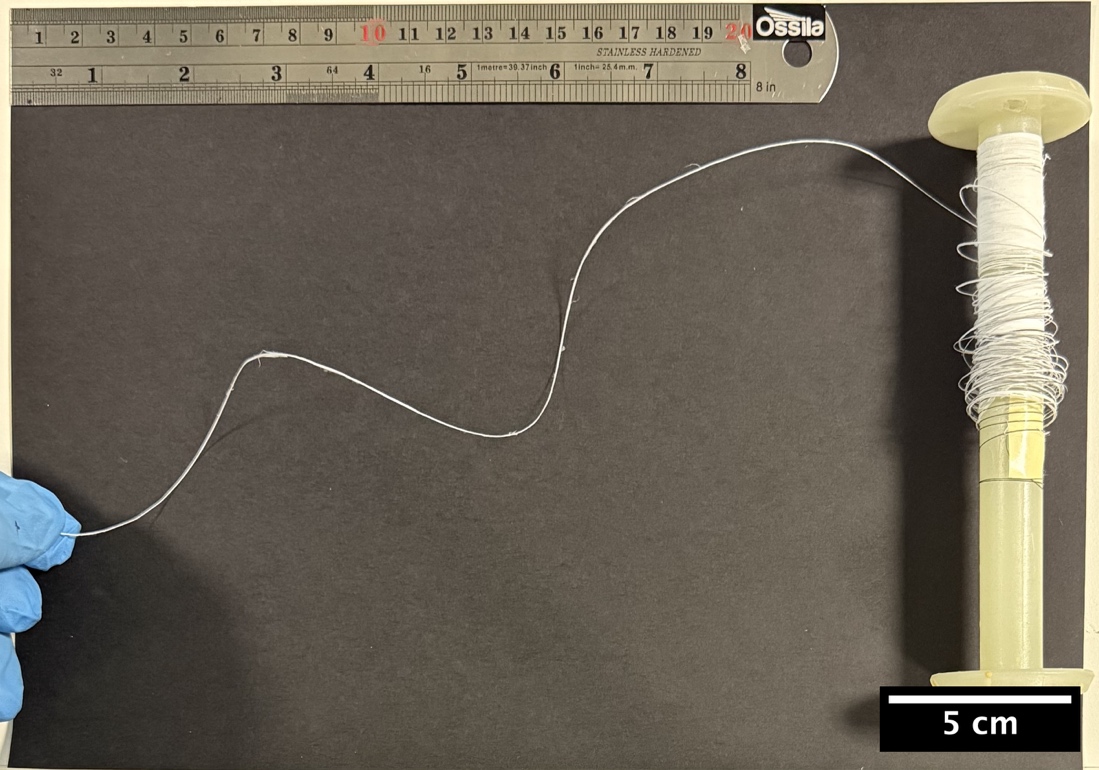


**Figure S9.** Photograph of the PAN-MXene yarns collected on the mandrel spool after the yarn electrospinning process.
